# Single-cell transcriptome analysis reveals immunosuppressive landscape in overweight and obese colorectal cancer

**DOI:** 10.1186/s12967-024-04921-5

**Published:** 2024-02-04

**Authors:** Guozhong Xiao, Yihui Zheng, Huaxian Chen, Minyi Luo, Chaoxin Yang, Donglin Ren, Pengfei Qin, Heng Zhang, Hongcheng Lin

**Affiliations:** 1 Department of General Surgery (Department of Coloproctology), The Sixth Affiliated Hospital, Sun Yat-Sen University, Guangzhou, 510655 China; 2 BGI Research, Shenzhen, 518083 China; 3 Guangdong Provincial Key Laboratory of Colorectal and Pelvic Floor Diseases, The Sixth Affiliated Hospital, Sun Yat-Sen University, Guangzhou, 510655 China; 4 Biomedical Innovation Center, The Sixth Affiliated Hospital, Sun Yat-Sen University, Guangzhou, 510655 China; 5 BGI Research, Chongqing, 401329 China

**Keywords:** Colorectal cancer, Overweight, Obesity, Obese, Single-cell RNA sequencing, Tumor microenvironment, Immunosuppressive

## Abstract

**Background:**

Overweight and obesity are established risk factors for various types of cancers including colorectal cancer (CRC). However the underlying molecular mechanisms remain unclear. An in-depth understanding of the oncologic characteristics of overweight and obese CRC at the single-cell level can provide valuable insights for the development of more effective treatment strategies for CRC.

**Methods:**

We conducted single-cell RNA sequencing (scRNA-seq) analysis on tumor and adjacent normal colorectal samples from 15 overweight/obese and 15 normal-weight CRC patients. Immunological and metabolic differences between overweight/obese CRC and non-obese CRC were characterized.

**Results:**

We obtained single-cell transcriptomics data from a total of 192,785 cells across all samples. By evaluating marker gene expression patterns, we annotated nine main cell types in the CRC ecosystem. Specifically, we found that the cytotoxic function of effector T cells and NK cells was impaired in overweight/obese CRC compared with non-obese CRC, relating to its metabolic dysregulation. CD4^+^T cells in overweight/obese CRC exhibited higher expression of immune checkpoint molecules. The antigen-presenting ability of DCs and B cells is down-regulated in overweight/obese CRC, which may further aggravate the immunosuppression of overweight/obese CRC. Additionally, dysfunctional stromal cells were identified, potentially promoting invasion and metastasis in overweight/obese CRC. Furthermore, we discovered the up-regulated metabolism of glycolysis and lipids of tumor cells in overweight/obese CRC, which may impact the metabolism and function of immune cells. We also identified inhibitory interactions between tumor cells and T cells in overweight/obese CRC.

**Conclusions:**

The study demonstrated that overweight/obese CRC has a more immunosuppressive microenvironment and distinct metabolic reprogramming characterized by increased of glycolysis and lipid metabolism. These findings may have implications for the development of novel therapeutic strategies for overweight/obese CRC patients.

**Supplementary Information:**

The online version contains supplementary material available at 10.1186/s12967-024-04921-5.

## Background

Colorectal cancer (CRC) is a common malignant tumor of the digestive tract. Worldwide, the incidence and mortality of CRC are at the forefront of malignant tumor diseases. The Global Cancer Burden Report shows that the global incidence of CRC ranked third among malignant tumors in 2020 [1]. Extensive epidemiological and clinical studies have provided evidence that overweight and obesity is a risk factor for developing CRC [2]. While there is growing evidence indicating a link between overweight, obesity, and CRC, the precise molecular mechanisms underlying this association are still not fully understood [3]. Furthermore, there has been a notable rise in the incidence of early-onset CRC associated with overweight and obesity in recent years [4, 5]. For most tumors, chemotherapy is an important and most common treatment option for cancer treatment [6]. Chemotherapy also plays a key role in the treatment of advanced CRC. However, recent studies have shown that obesity promotes chemoresistance by inducing chronic inflammation, altering pharmacokinetics, and altering tumor-associated adipokine secretion [7]. Moreover, overweight and obese CRC is linked to a worse prognosis [8–10]. Of particular interest, obesity rates are increasing globally. Obesity has become a major public health issue in China where 34.3% of adults face issues of overweight, and 16.4% contend with obesity [11]. Notably, the sustained or escalating presence of overweight or obese Body Mass Index (BMI) over time is correlated with an increased susceptibility to gastrointestinal cancer [12]. Consequently, it is crucial to reveal the mechanism by which obesity increases tumor burden.

In the process of CRC development, certain epigenetic mechanisms such as DNA methylation, non-coding RNA regulation, and histone modifications play critical roles [13–15]. Previous studies have indicated that obesity can alter the DNA methylation profile in the colon of mice, thereby promoting the development of CRC. Additionally, it is widely recognized that tumors induce significant metabolic changes in the host [16]. Increasing researches indicate that obesity-induced abnormal lipid metabolism, adipokines and hormones, chronic inflammation, gut dysbiosis, and disruption of bile acid homeostasis may play important roles in the complex metabolic regulation of CRC tumorigenesis [3, 17]. Previous studies have also found that obesity drives epigenomic changes in the colon epithelium and reprograms cell metabolism in the colon epithelium to promote CRC tumor initiation and progression [18–20]. Meanwhile, recent studies have shown that obesity may also indirectly promote tumorigenesis by suppressing anti-tumor immunity. Yamada et al.’s study [21] found that reduced CD4^+^T cell numbers and dysfunction in obese CRC mouse models lead to reduced anti-tumor responses of CD4^+^ and CD8^+^T cells, ultimately accelerating the progression of CRC. Ringel et al. [22] used single-cell sequencing technology to analyze high-fat diet induced obesity mouse tumor models and found that obesity reshaped the overall metabolism of tumor-infiltrating immune cells, reduced the number of important immune cells CD8^+^T cells in tumors and weakened their anti-tumor activity. These results indicate that obesity affects the number and function of immune cells in the tumor microenvironment (TME). The TME compositions including various immune cells and nonimmune stromal cells play determinant roles in tumor growth or decline [23]. However, there are currently few studies reporting the impact of obesity on the cell number and function of the TME in CRC, and it is also unclear how obesity affects the interaction between tumors and immune cells in the TME. Single-cell RNA sequencing (scRNA-seq) reveals the highly complex cellular composition of the TME at high resolution and can also describe the complex interactions between cancer cells and the microenvironment. However, a comprehensive description of the TME of overweight and obese CRC at the single-cell resolution level is still lacking.

This study aimed to explore the characteristics of the TME of overweight and obese CRC patients and compare their immune and metabolic profiles with non-obese CRC patients, which may help design more effective CRC treatment strategies. We performed scRNA-seq analysis on tumor and adjacent normal colorectal samples from overweight/obese and normal-weight CRC patients. We provide some new insights into the molding of tumor cells into their surrounding circumstances and the metabolic preferences of tumor cells from different weight groupings. Furthermore, our study also revealed compositional and functional differences in immune cell and stromal cell subpopulations in overweight/obese CRC. In conclusion, our study is expected to provide some attractive clues and supporting evidence for the treatment of overweight and obese CRC patients.

## Methods

### Patient recruitment and ethical approval

Thirty patients with histologically confirmed CRC were enrolled in this study. BMI was calculated as a patient's weight in kilograms, divided by height squared in meters and was further categorized using the Working Group on Obesity in China recommended BMI criteria [11, 24]: normal weight as 18.5–24 kg/m^2^, overweight as 24–28 kg/m^2^, and obesity as ≥ 28 kg/m^2^. Then, we classified both overweight and obese CRC patients into the obese group and patients with normal weight into the non-obese group, to evaluate the effect of excess body weight. There were 14 tumor samples were obtained from the obese patients and 15 tumor samples from the non-obese patients. In addition, adjacent normal mucosa tissue samples (located > 3 cm from the tumor) were taken as the normal control group, including two samples from the obese patients and three samples from the non-obese patients. The clinical characteristics of all samples are shown in Additional file 1: Table S1. All sampling and experimental procedures were approved by the Ethics Committee of the Sixth Affiliated Hospital of Sun Yat-sen University (license no. 2023ZSLYEC-403).

### Single-cell suspensions, library construction, and sequencing

Fresh tumor tissue samples were washed with Dulbecco’s phosphate-buffered saline (DPBS) and cut into 1–2 mm^3^ slices on ice. The samples were subsequently enzymatically digested using the MACS human tumor dissociation kit (Miltenyi Biotec). The single-cell 3’-libraries were constructed using the DNA Nanoball (DNB) elab C4 scRNA Preparation Kit according to the manufacturer's instructions. Libraries were sequenced on the DNBelab C4 platform, and then the raw sequencing reads were filtered and demultiplexed using PISA (https://github.com/shiquan/PISA).

### Single-cell RNA-seq data processing

The filtered reads were aligned to the human genome using STAR (v.2.7.4a) and sorted using Sambamba (v.0.7.0). The cell-gene count matrix was converted to a Seurat object using the R Seurat package (v.4.1.0) for downstream analysis. To remove low-quality cells, the following thresholds were applied: the number of genes identified in individual cells ranged from 300 to 6000, and the percentage of mitochondrial gene expression in individual cells was < 20%. The expression matrix was normalized to account for differences in sequencing depth per cell for each sample with the “NormalizeData” function in the Seurat package (v.4.3.0) [25]. Then, the Seurat “FindVariableFeatures” function was used to generate highly variable genes, a total of 3000 highly variable genes were selected based on normalized dispersion. RunPCA and RunUMAP functions implemented in Seurat were also used to reduce dimensionality. We used the “FindClusters” function in Seurat on the top 25 principal components with resolution 0.4 to perform the first-round cluster and annotation.

### Determination of cell type and identification of differentially expressed genes

The identification of differentially expressed genes (DEGs) within each cell cluster was carried out using the “FindAllMarker” function provided by Seurat, employing default parameters. The annotation of cell types and subtypes was determined based on the expression of known canonical marker genes associated with respective cell types. To prevent excessive classification, raw clusters with similar gene expression patterns were merged. The “FindMarkers” function, using default parameters provided by Seurat, was utilized to identify DEGs between different groups or clusters.

### Tissue distribution of cell types

To quantify the tissue preference of each cluster, we followed the methodology outlined in the previous study [26]. Specifically, we calculated the ratio of observed to expected cell numbers (Ro/e) for each cell type across different tissues. A Ro/e value greater than 1 indicates an enrichment of the particular cell type in the corresponding tissue.

### Pathway analysis

The gene ontology (GO) analysis on DEGs in this study was performed by the clusterProfiler package (v.4.2.2) [27]. Gene sets with a p-value of < 0.05 were considered to be significantly enriched. Gene Set Enrichment Analysis (GSEA) was used to investigate biological states or function differences of cells from obese/non-obese CRC [28]. Gene set variation analysis (GSVA) was conducted with the GSVA package (v.1.42.0) [29]. The gene sets of hallmarks and KEGG pathways used in this study were acquired from the molecular signature database [30]. The detailed list of genes set for GSVA score of cells and their reference sources were provided in Additional file 2: Table S2.

### Metabolic pathway analysis

The analysis of the metabolic pathways was performed using the scMetabolism R package with parameters “method = VISION, metabolism.type = KEGG” [31]. The activity difference of KEGG metabolic pathways between groups was measured by two-sided Wilcoxon rank-sum test.

### Developmental trajectory analysis

Developmental trajectory analysis was performed using Monocle 2 package [32]. First, the DEGs of eight fibroblast cell subtypes were identified and subjected to Monocle analysis. Then, dimensionality reduction and visualization were performed using “DDRTree” and “plot_cell_trajectory” functions in the Monocle 2 package. Genes that changed along the identified trajectory were identified by performing a likelihood ratio test using the function “differentialGeneTest” in the Monocle 2.

### Cell–cell interaction analysis

To investigate the cell–cell communications mediated by ligand-receptor interaction between different cell types, we used CellChat to analyze and compare the cell–cell communications between different groups [33]. By using CellChat with recommended parameters, the CellChat algorithm was then run to calculate the probable interactions and pathways via the “computeCommunProb” and “computeCommunProbPathway”. We also run the “filterCommunication” to filter out interactions with less than 10 cells in each cell type.

### Survival analysis

Bulk RNA-seq data along with the curated clinical data from patients with colon cancer and rectal cancer were obtained from the UCSC Xena database website (http://xena.ucsc.edu/). To evaluate the prognostic effect of gene sets derived from specific cell clusters, GSVA analysis was applied to calculate the combined expression value of the cell type–specific signatures. All signatures are ranked according to their fold change values and we used the top 10 genes for each subset in survival analysis of the cancer genome atlas (TCGA) data. The optimal low/high cutoffs for the expression level of these marker genes were assessed by the “surv_cutpoint” function in the survminer R package. Then, the survival curves were plotted using the “ggsurvplot” function in the survminer.

### InferCNV analysis to identify malignant cells

The InferCNV package (https://github.com/broadinstitute/inferCNV) was used to infer CNVs in epithelial cells and to recognize cancer cells. Stromal cells in each sample were used as the reference for determining somatic variants. For the inferCNV analysis, the following parameters were used: “denoise,” and a value of 0.1 as the “cutoff” value. Epithelial cells with relatively higher CNV scores were considered malignant cells.

### Immunohistochemistry

Tumor tissues were fixed with 10% formaldehyde and embedded in paraffin. Sections were subjected to immunohistochemical (IHC) staining according to standard procedures. We performed the IHC by using the *SCD* antibody (Proteintech, 28678-1-AP, 1:800) and *LDHA* antibody (Proteintech, 19987-1-AP, 1:200). The primary antibody was incubated at 4 ℃ overnight. Biotinylated secondary antibody was then added and incubated for 20 min at 37 ℃. Staining intensity of *SCD* and *LDHA* of 20 overweight/obese CRC and 20 non-obese CRC samples were then quantified. For each slide, three non-overlapping fields of view for each histologic region were randomly captured at 20 × magnification, and the staining intensity of *SCD* and *LDHA* was finally semi-quantified using the Image J software by transforming it into mean optical density. The statistical difference in staining intensity of *SCD* and *LDHA* between overweight/obese CRC and non-obese CRC was determined by the unpaired two-tailed Student’s t-test.

### Statistical analysis

The gene expression or gene signature was compared between the two groups of cells using an unpaired two-tailed Student’s t-test. We investigated the correlation of scores between different cell subsets. Scatter plots and Spearman correlation analysis were conducted using ggscatter in the ggplot2 package. These correlations were examined across all CRC samples. Samples with missing values were excluded from the analysis. All statistical analyses and data presentations were performed by the R program (v.4.1.0). Statistical significance was set at a *P*-value of < 0.05. Significance levels are indicated as *P < 0.05, **P < 0.01 and ***P < 0.001.

## Results

### Single-cell transcriptome landscape in CRC

**Fig. 1:**
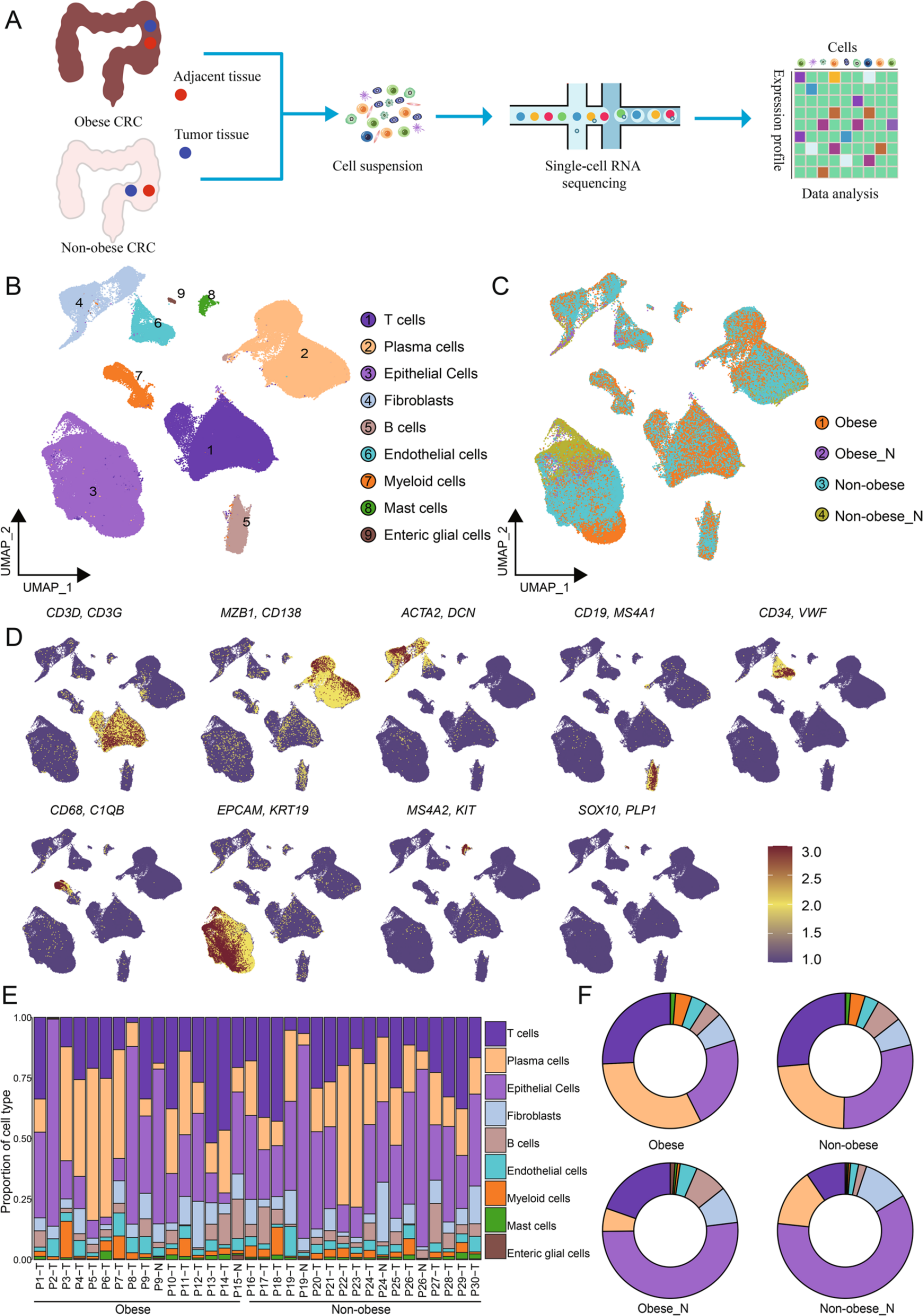
Single cell transcriptome landscape of CRC patients.** A** Schematic workflow of experimental design and data analysis in this study. **B** UMAP plot of the cell types, colored by cell types. **C** UMAP plot of the groups, colored by different groups. **D** Log normalized expression of marker genes of each cell type. **E** Proportions of each cell type in each sample, colored by cell types. F Proportions of each cell type in different groups, colored by cell types

To explore the cellular diversity of overweight/obese CRC, we integrated scRNA-seq data from 14 overweight/obese CRC and 15 non-obese CRC tumor samples, categorizing them into obese and non-obese CRC (Obese and Non-obese) groups (Fig. 1A). We also conducted scRNA-seq analysis on adjacent normal tissue obtained from two overweight/obese CRC samples and three non-obese CRC samples, categorizing them into normal obese (Obese_N) and non-obese CRC (Non-obese_N) groups. The clinical characteristics of these samples are shown in Additional file 1: Table S1. Considering that some clinicopathological features such as age, sex, and tumor stage may be related to the immune microenvironment and metabolism of patients, we collected the clinical characteristics of all overweight/obese CRC and non-obese CRC samples, the results indicated that there were no significant differences in terms of age, sex, and clinical stage between the obese CRC group and the non-obese CRC group, as shown in Additional file 1: Table S1. This is important as it helps ensure that these factors do not confound the comparison between the two groups. Following rigorous quality control steps (Method section), we obtained single-cell transcriptomics data from a total of 192,785 cells across all samples for subsequent analysis. By evaluating marker gene expression patterns, we annotated nine main cell types in the CRC ecosystem, including epithelial cells, T cells, B cells, plasma cells, myeloid cells, mast cells, fibroblasts, endothelial cells, and enteric glial cells. These cell types distributed across both the obese and non-obese samples (Fig. 1B–D). Notably, significant differences in the abundance of cell types were observed between the different groups, indicating distinct microenvironmental components between obese CRC and non-obese CRC samples (Fig. 1C). While all cell types presenting in all samples, the proportion of each cell type varied across the samples (Fig. 1E, Additional file 3: Fig.  S1A). The relative abundance of the nine major cell types in all samples and four groups is shown in Fig. 1E, F. Our results revealed that immune cells, including plasma cells, myeloid cells, and T cells, were more abundant in tumor samples, whereas fibroblasts, epithelial cells, and enteric glial cells were enriched in adjacent normal tissues (Additional file 3: Fig. S1B). Importantly, obese tumor samples showed a higher proportion of plasma cells and a lower proportion of B cells compared to non-obese tumor samples (Additional file 3: Fig. S1B). In summary, our results indicate significant differences in the composition of cell types between tumor tissues and adjacent normal tissues in CRC. Moreover, our findings highlight that obese CRC samples have a distinct tumor microenvironment compared to non-obese CRC.

### Different functional characteristics of T&NK cell subpopulations between obese CRC and non-obese CRC

**Fig. 2:**
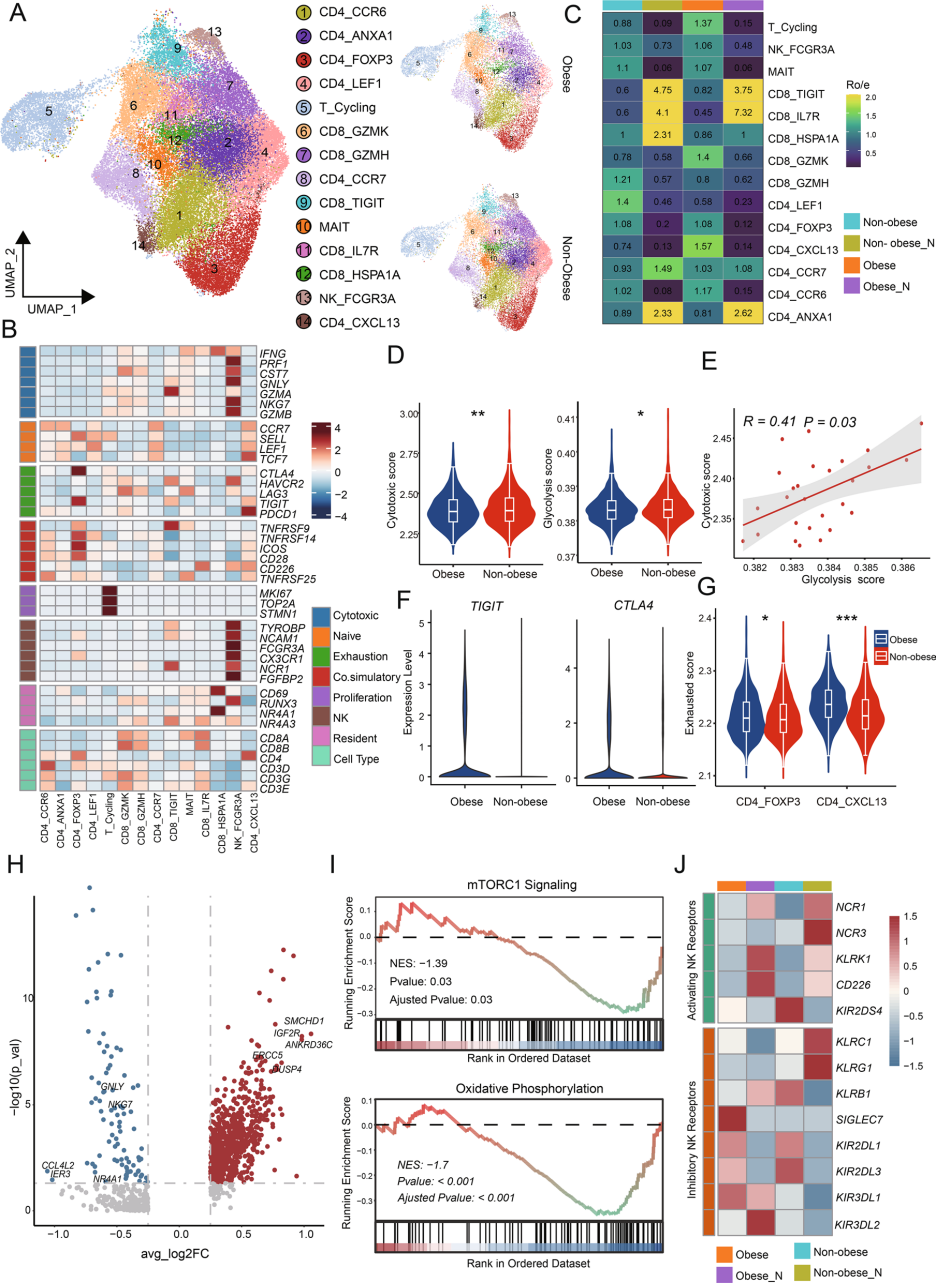
Functional and metabolic dysregulation of T&NK cell subpopulations in obese CRC. **A** UMAP plots of the T cell subsets, colored by cell subsets. **B** Heatmap showing the expression of canonical T/NK cell marker genes. **C** Tissue prevalence of each T cell subset estimated by Ro/e score, in which Ro/e denotes the ratio of observed to expected cell number. **D** Signature scores for CD8^+^GZMK^+^T cells. Comparisons were performed by unpaired two-tailed Student’s t-test. Significance levels are expressed as *P < 0.05, **P < 0.01 and ***P < 0.001. **E** The Spearman correlation analysis between the scores of cytotoxicity score and glycolysis metabolism score in CRC samples. **F** Violin plots showing immune checkpoints expression of CD4^+^T cells from obese CRC and non-obese CRC samples. **G** Exhaustion signature scores for CD4^+^FOXP3^+^T and CD4^+^CXCL13^+^T cells. Comparisons were performed by unpaired two-tailed Student’s t-test. Significance levels are expressed as *P < 0.05, **P < 0.01 and ***P < 0.001. **H** Volcano plot showing the DEGs of NK cells in obese CRC and non-obese CRC. Red and blue represent DEGs that are up—and down-regulated in obese CRC, respectively. **I** GSEA analysis comparing transcriptomic profiles of NK cells in obese CRC and non-obese CRC, with positive scores indicating enrichment in the obese CRC group, and negative scores indicating enrichment in the non-obese CRC group. NES, normalized enrichment score. **J** Heatmap showing the expression of NK cells activating and inhibitory receptors

T&NK lymphocytes were identified as the most abundant TME immune cell populations of CRC (Fig. 1E). To gain insights into the functional subtypes of T cell populations in CRC, we performed unsupervised cluster analysis using extracted T cells from all samples, including six subsets of CD4^+^T cells, five subsets of CD8^+^T cells, one NK cell subset, one mucosal associated invariant T cell subset (MAIT) and a proliferative T cell subset (Fig. 2A). Each cluster was defined by the expression of specific marker genes (Additional file 4: Fig. S2A). The CD4^+^T cells were further categorized into CCR6^+^T cells, ANXA1^+^T cells, FOXP3^+^regulatory T cells (Treg), LEF1^+^naïve T cells, CCR7^+^naïve T cells, and CXCL13^+^T cells (Fig. 2A, Additional file 4: Fig. S2A). Previous studies have shown that enrichment of CXCL13^+^T cells, which express high levels of *PDCD1* and *CTLA4*, are associated with enhanced sensitivity to immunotherapy targeting *PD1* or *CTLA4* in CRC patients [26]. CD8^+^T cells were divided into GZMK^+^T cells, GZMH^+^T cells, TIGIT^+^T cells, IL7R^+^T cells, and HSPA1A^+^T cells (Fig. 2A, Additional file 4: Fig. S2A). Notably, HSPA1A^+^T cells also exhibited high expression of genes related to tissue-resident characteristics, implying their potential as tissue-resident T cells (Fig. 2B). The FCGR3A^+^NK cells showed high expression of cytotoxic genes such as *GNLY*, *GZMB*, and *PRF1*, as well as the inhibitory-related gene *HAVCR2* (Fig. 2B). The high expression of *FCGR3A* suggests that these cells might be recruited from the peripheral blood. Importantly, we observed that these T cell subtypes were present in both obese and non-obese samples, as well as among different patients. However, there were notable differences in their distribution. Specifically, the majority of CD8^+^T cell clusters were significantly enriched in adjacent normal tissue samples, whereas CD4^+^T cell clusters including CXCL13^+^T cells and Treg cells showed higher enrichment in tumor samples (Fig. 2C). Interestingly, the distribution of CXCL13^+^T cells was particularly prominent in the obese tumor samples. Additionally, FCGR3A^+^NK cells were enriched in tumor samples, indicating that tumor-infiltrating NK cells primarily originated from peripheral blood (Fig. 2C). These findings suggest potential differences in T cell functionality between obese CRC and non-obese CRC patients.

We conducted further analysis to examine the differences in pathways and gene expression among T cell subsets. Specifically, we compared the cytotoxic scores of all CD8^+^T cells and found that GZMK^+^T cells exhibited the highest cytotoxic scores, indicating their importance as key effector cells in the immune response against tumors (Additional file 4: Fig. S2B). Interestingly, GZMK^+^T cells in obese tumor samples showed a lower cytotoxic expression pattern compared to those in non-obese tumor samples (Fig. 2D), which suggests that obesity may impact the cytotoxic function of GZMK^+^T cells in CRC. Obesity is known to cause chronic metabolic dysfunction, resulting in altered levels of insulin/glucose, leptin/adiponectin, and other hormones and adipokines in the blood [34–36]. Previous studies have also shown that obesity and the metabolic dysfunctions associated with it can have a profound effect on the status of the immune system [37]. In addition, immune cells undergo dynamic metabolic changes during development and activation [38, 39]. Therefore, we further investigated the metabolic activity of T cells. Our findings revealed that GZMK^+^T cells in obese tumor samples have a lower glycolytic metabolic score compared to those in non-obese tumor samples (Fig. 2D). Naïve T cells remain in a quiescent state, reliant on oxidative phosphorylation for their energy needs. However, upon activation, their metabolic profiles shift to support increased glycolysis and meet the energy demands of the activated cell. We investigated the correlation between the cytotoxic scores and glycolysis metabolism scores of GZMK^+^T cells in obese tumor samples and non-obese tumor samples (Fig. 2E), and the results showed that the cytotoxic scores and glycolysis scores of GZMK^+^T cells were positively correlated in CRC samples. These results suggest that decreased effector T cells cytotoxic activity in obese CRC samples may be related to impaired glycolysis metabolism. Subsequently, we compared the expression of exhaustion related molecules in CD4^+^T cells and found that CD4^+^T cells in obese tumor samples had higher expression of *TIGIT* and *CTLA4* molecules compared with non-obese tumor samples (Fig. 2F). CXCL13^+^T cells were found to have a higher expression level of *PDCD1* in obese CRC (Additional file 4: Fig. S2C), which implied that obese CRC may have a better response to anti-PD-1 treatment. We further compared the exhaustion scores of CD4^+^T cells and showed that Treg cells and CXCL13^+^T cells had higher exhaustion scores in obese tumor samples (Fig. 2G). These results may indicate that obese CRC induces a T cell exhaustion phenotype. It is characterized by increased expression of inhibitory receptors.

We analyzed the differential gene expression in NK cells between obese CRC and non-obese tumor samples. The results showed that the cytotoxicity-related markers *GNLY* and *NKG7* were down-regulated in NK cells of obese CRC compared with non-obese CRC (Fig. 2H). This indicate that NK cell killing function is down-regulated in obese CRC. Cellular metabolism also plays a crucial role in NK cell function. We used GSEA to compare the different pathways in NK cells between obese tumor samples and non-obese tumor samples. NK cell mTORC1 pathway and oxidative phosphorylation pathway activity were decreased in obese CRC (Fig. 2I). Previous studies have shown that the mTOR pathway and oxidative phosphorylation activation are critical for the NK cell cytotoxicity process [40, 41]. Therefore, our study may indicate NK cell dysfunction in obese CRC due to decreased metabolism of mTOR and oxidative phosphorylation. NK cells express a variety of receptors that coordinate their effector function. To further investigate the differences expression in functional receptors between non-obese tumor samples and obese tumor samples, we analyzed the expression of these receptors. Our results demonstrated that most of the activating receptors were less expressed in NK cells of obese CRC, while the inhibitory receptor *SIGLEC7* showed significantly high expression in obese CRC (Fig. 2J). *SIGLEC7*, which belongs to the sialic acid-binding immunoglobulin-like lectins (SIGLECS) family predominantly expressed in leukocytes, has been previously identified as a potential immune checkpoint in bladder cancer [42]. It has been shown to dampen the cytotoxic activity of NK cells against bladder cancer cells. Given its significantly high expression in obese CRC, *SIGLEC7* may represent a promising novel immune checkpoint for this particular subset of CRC patients.

Collectively, our data suggest that effector T cells and NK cells in the obese CRC TME are dysfunctional and have lower cytotoxicity, which may both be associated with dysregulated metabolic activity. In contrast, CD4^+^T cells in the TME of obese CRC had a higher exhaustion score and expressed higher exhaustion genes.

### Metabolic reprogramming of macrophages and functional suppression of DCs in obese CRC

**Fig. 3:**
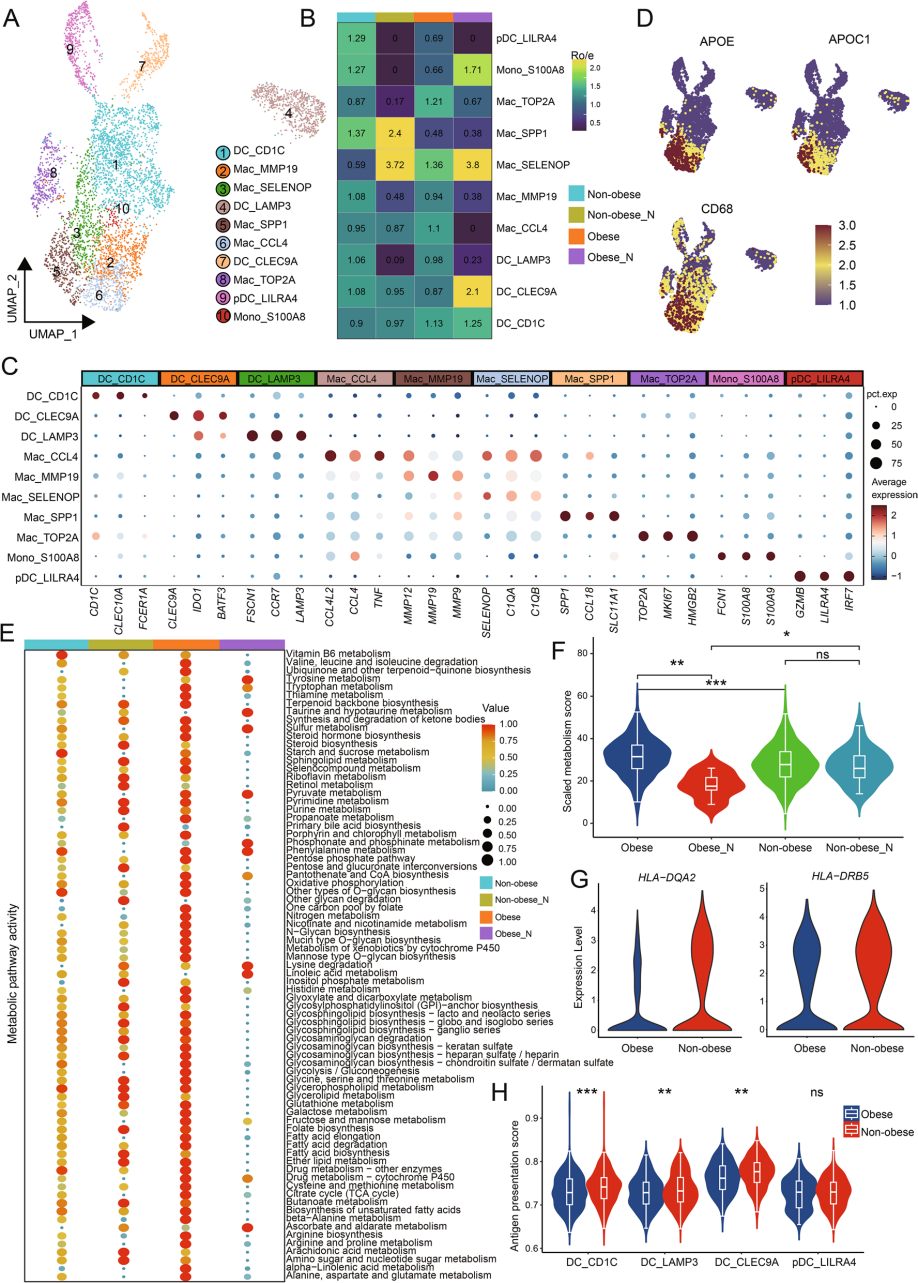
The immunosuppressive phenotype of obese CRC-derived myeloid cells. **A** UMAP plots of the myeloid cell subsets, colored by cell subsets. **B** Tissue prevalence of each myeloid cell subset estimated by Ro/e score, in which Ro/e denotes the ratio of observed to expected cell number. **C** The expression of the marker genes of the myeloid cell subsets. The pct.exp reflects the percentage of cells expressing the gene at non-zero levels. The Average expression reflects the averaged log-normalized expression. **D** Log-normalized expression of selected marker genes in myeloid cells. **E** Dot plots showing the metabolic activity analysis of three lipid-associated macrophages (LAMs) subsets by scMetabolism. The circle size and color darkness both represent the scaled metabolic score. **F** Violin plots showing the metabolic score of metabolic pathways in three LAMs subsets. Comparisons were performed by unpaired two-tailed Student’s t-test. Significance levels are expressed as *P < 0.05, **P < 0.01 and ***P < 0.001. **G** Violin plots showing MHC class II molecules expression of DC from obese CRC and non-obese CRC samples. **H** Violin plots showing the antigen presentation score in four DCs subsets. Comparisons were performed by unpaired two-tailed Student’s t-test. Significance levels are expressed as *P < 0.05, **P < 0.01 and ***P < 0.001

The proportion of myeloid cells in the tumor tissue was significantly higher than that in the adjacent normal tissue (Additional file 3: Fig. S1B). Subsequently, we investigated the composition and gene expression of myeloid cells in non-obese and obese group. Reclustering of all myeloid cells extracted revealed 10 cell subsets with different frequencies in different tissues (Fig. 3A, B). The monocyte population (cluster 10) was characterized by high expression of *FCN1*, *S100A8*, and *S100A9* (Fig. 3C). For macrophages we used marker genes based on specific expression to define including MMP19^+^, SELENOP^+^, SPP1^+^, CCL4^+^macrophage, and a group of proliferative macrophages with high expression of proliferation genes *TOP2A* and *MKI67* (Fig. 3A, C). By calculating M1 and M2 polarization scores using relevant gene sets, we could not clearly distinguish M1 from M2 macrophages (Additional file 5: Fig. S3A), similar to a previous study [43]. Notably, SPP1^+^macrophages showed higher M2 characteristics and significant angiogenesis characteristics. CCL4^+^macrophages have higher M1 characteristics but also highly express M2 characteristics (Additional file 5: Fig. S3A). In summary, our findings highlight the limitations of the in vitro polarization model and suggest a more diverse and complex phenotype of macrophages within the TME. Furthermore, we identified four distinct subsets of dendritic cells (DCs), including LAMP3^+^DCs, which have been previously reported as tumor-specific and were found to be more enriched in tumor samples in our dataset [44]. We also observed a population of CD1C^+^DCs that exhibited high expression of *CD1C* and *CLEC10A*, resembling the previously reported DC2 cells [43]. Additionally, we identified a subset of CLEC9A^+^DCs expressing the classic DC1 cell marker gene, *CLEC9A*, predominantly distributed in obese adjacent tissues (Fig. 3B, C). Moreover, our analysis revealed a population of plasmacytoid dendritic cells (pDCs) characterized by high expression of *LILRA4* and *GZMB* (Fig. 3C).

Our analysis revealed three distinct lipid-associated macrophages (LAMs) exhibiting high expression of the macrophage marker *CD68* and lipid metabolism genes such as *APOE* and *APOC1* (Fig. 3D). Interestingly, one group of LAMs displayed elevated expression of *SPP1*, which has been reported to be secreted by tumor-associated macrophages (TAMs) to promote cancer progression [45]. On the other hand, the other two groups exhibited high expression of *CCL4* and *SELENOP*, both of which have immunosuppressive and tumor-promoting functions (Fig. 3C) [46, 47]. To gain further insights into the metabolic activity of LAMs in non-obese and obese samples, we used the scMetabolism package to systematically quantify metabolic activity using our scRNA-seq data. We calculated metabolic pathway activity scores for all KEGG metabolic pathways annotated in scMetabolism. Intriguingly, we discovered significantly higher metabolic scores in LAMs from obese tumor tissues compared to non-obese tumor and adjacent normal tissues (Fig. 3E, F), indicating that they are extremely active and vibrant in obese CRC. Notably, numerous lipid metabolism-related pathways, including fatty acid elongation and fatty acid degradation, were highly enriched in LAM cells infiltrating obese CRC (Fig. 3E).

DCs exhibit high expression of MHC class II molecules, such as *HLA-DRA* which are critical for activating T cells and promoting antitumor immunity. In our comparison of differentially expressed genes in DCs between non-obese tumor samples and obese tumor samples, we observed significantly lower expression of certain MHC class II molecules, such as *HLA-DQA2* and *HLA-DRB5*, in obese CRC compared to non-obese CRC (Fig. 3G). Furthermore, we examined the antigen presentation function of all DC subsets and found that, except for LILRA4^+^pDCs the antigen presentation function of DCs was significantly downregulated in obese CRC (Fig. 3H). DCs excel at antigen presentation and play a crucial role in initiating T cell-mediated antitumor immune responses. Therefore, we evaluated the correlation between the cytotoxic scores of GZMK^+^T cells and the antigen presentation scores of DCs in tumor samples, and the results showed a significant positive correlation (Additional file 5: Fig. S3B). This may imply that the ability of DCs to activate T cells is limited in obese CRC.

Taken together, these findings suggest that both macrophages and DCs play important roles in the establishment of an immunosuppressive TME in obese CRC.

### Functional characteristics of stromal cells in obese CRC

**Fig. 4:**
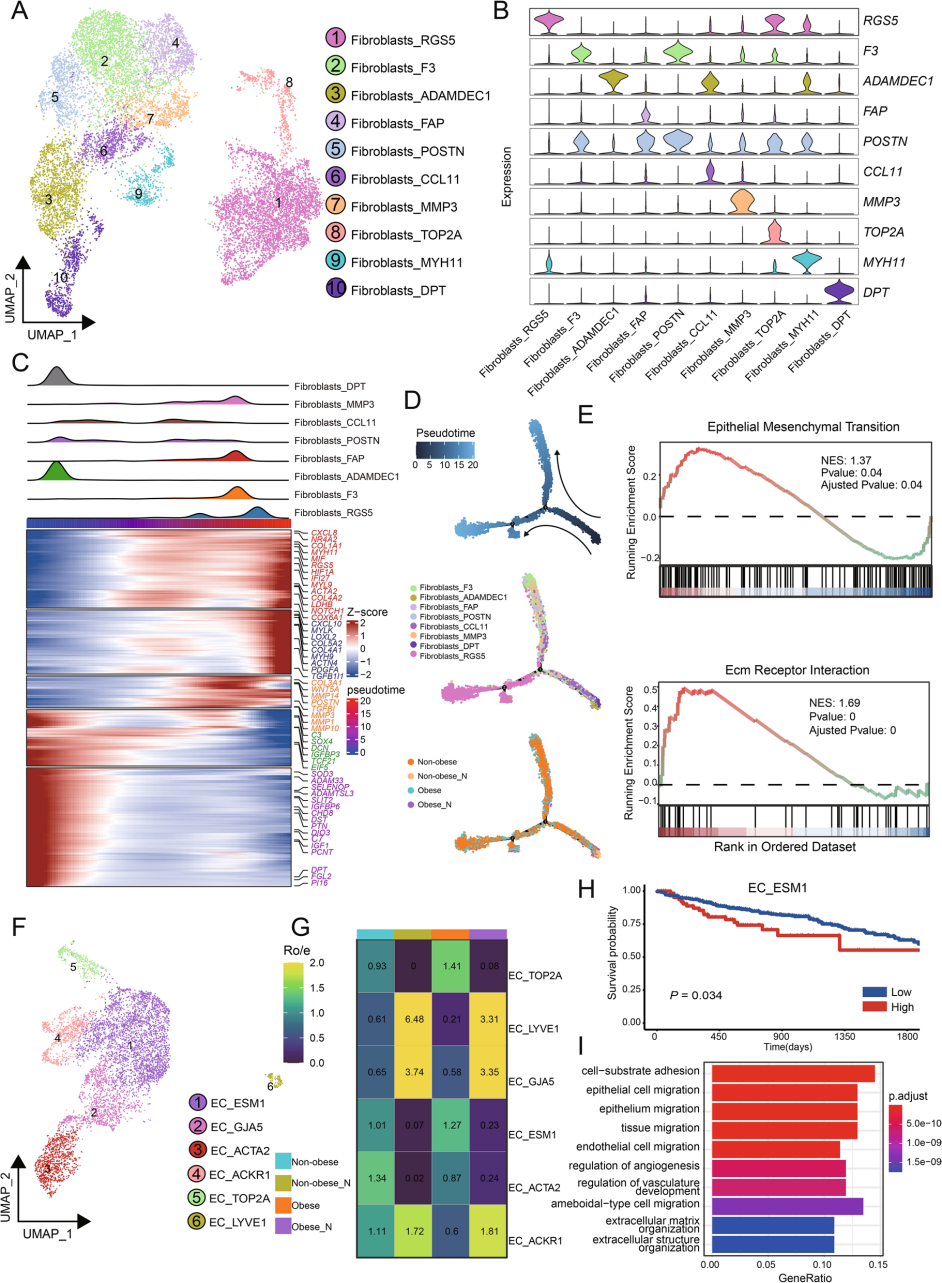
Dysregulation of stromal cells in obese CRC. **A** UMAP plots of the fibroblast subsets, colored by cell subsets. **B** Violin plots showing the expression of markers in various subsets of fibroblasts. **C** Heatmap showing the dynamic changes in gene expression along the pseudotime (lower panel). The distribution of fibroblasts subsets during the transition, along with the pseudo-time. Subsets are labeled by colors (upper panel). **D** Pseudotime-ordered analysis of fibroblasts. colored by pseudotime, cell subsets, and groups, respectively. **E** GSEA analysis comparing transcriptomic profiles of RGS5^+^fibroblasts in obese CRC and non-obese CRC, with positive scores indicating enrichment in the obese CRC group, and negative scores indicating enrichment in the non-obese CRC group. NES, normalized enrichment score. **F** UMAP plots of the endothelial cell subsets, colored by cell subsets. **G** Tissue prevalence of each endothelial cell subset estimated by the Ro/e score, in which Ro/e denotes the ratio of observed to expected cell number. **H** Kaplan–Meier survival analysis of TCGA CRC patients stratified by ESM1^+^EC. I Bar plots showing typically significantly enriched GO terms in ESM1^+^EC

Among the various types of stromal cells present in the TME, fibroblasts are the predominant component, and cancer-associated fibroblasts (CAFs) play significant and diverse roles in supporting tumor growth. We identified 10 distinct fibroblast subsets based on their specific expression markers (Fig. 4A). By analyzing the distribution of cell proportions (Additional file 6: Fig. S4A), we found that cells expressing ADAMDEC1^+^, DPT^+^, MYH11^+^, and POSTN^+^ markers were mainly derived from adjacent normal tissues, referred to as normal fibroblasts (NFs). Six tumor-rich clusters were considered CAF because they were primarily derived from tumor tissue (Fig. 4A, Additional file 6: Fig. S4A). There are several groups of CAFs that highly express inflammation genes and complement factors, consisting of four distinct subsets namely, F3^+^, FAP^+^, CCL11^+^, and MMP3^+^fibroblasts. Furthermore, there exists another subset characterized by RGS5^+^fibroblasts, which demonstrate elevated expression of classical myofibroblast marker, *ACTA2*, thus categorizing them as myofibroblasts (mCAFs) (Fig. 4B, Additional file 6: Fig. S4B). There was also a group of TOP2A^+^ fibroblasts with high expression of proliferation-related gene *TOP2A* (Fig. 4B). We analyzed major fibroblast subsets to explore potential activation processes specific to CAFs. Evolutionary trajectory analysis revealed two distinct activation pathways leading from NFs to CAFs. NFs derived from adjacent normal tissues were positioned at the initial stages of the trajectory. For CAF activation, there was an upregulation of expression in certain metal matrix proteases like *MMP3*, *MMP10*, and *MMP1*, while mCAF expressed high levels of genes involved in pathways crucial for cancer initiation and progression, including extracellular matrix remodeling genes (*COL1A1*, *COL4A2*) and genes involved in hypoxia regulation (*HIF1A*, *LDHB*) (Fig. 4C, D). Both hypoxia pathway scores and TGFB signaling pathway scores gradually increased along the CAF activation trajectory, reaching their peaks in the mCAF state (Additional file 6: Fig. S4C), indicating the important roles of hypoxia and TGFB pathway in CAF differentiation. The accumulation of RGS5^+^fibroblasts was significantly higher in tumor tissues compared to adjacent normal tissues (Additional file 6: Fig. S4A), highlighting the potential biological significance of RGS5^+^fibroblasts in the TME. Furthermore, we compared the pathway differences of RGS5^+^fibroblasts among different groups, and the results revealed the enrichment of pathways related to epithelial-mesenchymal transition and extracellular matrix receptor responses in obese CRC samples (Fig. 4E). These pathways have been implicated in tumor development and metastasis [48, 49].

Endothelial cells (ECs) play a crucial role in tumor angiogenesis, facilitating cancer growth and metastasis. Through unsupervised dimensionality reduction and clustering analysis, we identified six distinct subclusters of ECs with specific marker gene expression patterns: ESM1^+^EC, GJA5^+^EC, ACTA2^+^EC, ACKR1^+^EC, TOP2A^+^EC, and LYVE1^+^EC (Fig. 4F). Analyzing the relative proportions of each subcluster across the different patient groups revealed that ESM1^+^EC were enriched in the tumor samples, specifically in obese tumor samples (Fig. 4G). We then evaluated whether the abundance of the ESM1^+^EC subcluster had any association with patient survival using bulk RNA-seq data from TCGA. This showed that the frequency of ESM1^+^EC was negatively correlated with survival (Fig. 4H). The obese CRC samples demonstrates a increased enrichment of ESM1^+^EC cells, potentially indicating a correlation with a worse prognosis. To investigate the possible reasons why this subcluster exhibits poor prognosis in CRC, we performed the functional enrichment analysis. GO analysis revealed the enrichment of pathways related to the extracellular matrix (ECM) and the regulation of epithelial cell migration and adhesion in ESM1^+^EC (Fig. 4I), which are associated with tumor development and metastasis.

### Enrichment of IgG^+^plasma cells and dysregulation of B cell antitumor function in obese CRC

**Fig. 5:**
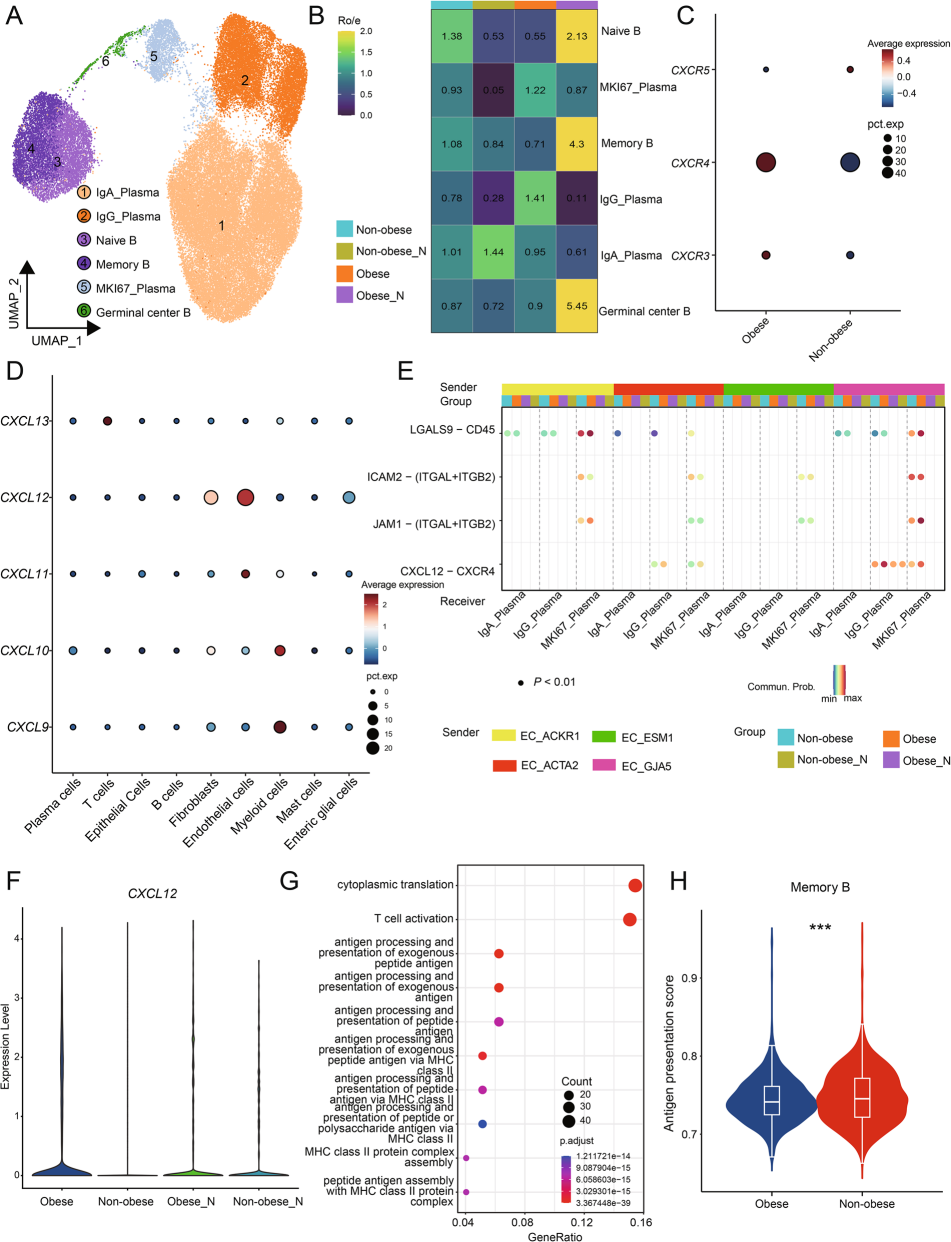
Enrichment of IgG plasma cells and immunosuppression of B cells in obese CRC. **A** UMAP plots of the B/plasma subsets, colored by cell subsets. **B** Tissue prevalence of each B/plasma cell subset estimated by the Ro/e score, in which Ro/e denotes the ratio of observed to expected cell number. **C** Dot plots showing the expression of the chemokine receptors in IgG^+^plasma cells. The pct.exp reflects the percentage of cells expressing the gene at non-zero levels. The Average expression reflects the averaged log-normalized expression. **D** Dot plots showing the expression of the chemokine in all cell types. The pct.exp reflects the percentage of cells expressing the gene at non-zero levels. The Average expression reflects the averaged log-normalized expression. **E** Dot plots showing the comparison of communication probabilities from four endothelial cell subsets to three plasma cell subsets among different groups. **F** Violin plots showing the expression of *CXCL12* in endothelial cell subsets, colored by groups. **G** Dot plots showing typically significantly enriched GO terms in memory B cells. **H** Violin plots showing the antigen presentation score in memory B cells. Comparisons were performed by unpaired two-tailed Student’s t-test. Significance levels are expressed as *P < 0.05, **P < 0.01 and ***P < 0.001

To further reveal the diversity of B cells and their immunological characteristics, we conducted detailed clustering analysis of B cells and identified three subsets of MS4A1^+^B cells along with three subsets of plasma cells (Fig. 5A). The plasma cells were further categorized into IgG^+^plasma cells, IgA^+^plasma cells, and proliferating plasma cells based on the expression of specific marker genes. Naïve B cells expressed *IGHD*, memory B cells expressed *TNFRSF13B* and *GPR183*, and germinal center B cells exhibited high expression levels of *BCL6* and *MKI67* (Additional file 7: Fig. S5A). In assessing the distribution of B cell plasma cell subsets across different tissues, we observed a higher enrichment of IgA^+^plasma cells in none-obese adjacent normal tissues (Fig. 5B). IgA antibodies are primarily found in mucosal areas such as the gastrointestinal tract, respiratory tract, and urogenital tract, where they play a critical role in preventing pathogen colonization. Conversely, IgG^+^plasma cells displayed a preferential enrichment in obese tumor samples (Fig. 5B). Previous studies have highlighted a correlation between the abundance of CXCR4^+^IgG^+^plasma cells and the severity of intestinal inflammatory diseases [50].

Then we first asked how IgG^+^ plasma cells were recruited into the obese CRC microenvironment. We examined chemokine receptor expression of IgG^+^plasma cells in obese tumor samples and non-obese tumor samples, including *CXCR3*, *CXCR4* and *CXCR5*, and found that IgG^+^plasma cells in obese CRC highly expressed CXCR4 (Fig. 5C). Next, we compared the expression levels of chemokines across all cell types and made an intriguing discovery: *CXCL12*, which is the ligand for *CXCR4*, was found to be most highly expressed in ECs (Fig. 5D). Based on this finding, we hypothesized that ECs might be involved in the recruitment of IgG^+^plasma cells. To investigate this further, we examined the cell–cell interactions between plasma cells and ECs using CellChat. The results revealed that the interaction via the *CXCL12-CXCR4* pathway was particularly strong in obese CRC samples (Fig. 5E). We also compared the expression of *CXCL12* in endothelial cells and found that endothelial cells in obese tumor samples exhibited the highest level of *CXCL12* expression (Fig. 5F), pointing towards the possibility that *CXCL12* secreted by endothelial cells in obese CRC could attract IgG^+^plasma cells expressing *CXCR4*.

GO enrichment analysis demonstrated that memory B cells shared similar functions with naïve B cells and were enriched in pathways related to antigen presentation and T cell activation (Fig. 5G). This suggests that memory B cells primarily contribute to anti-tumor immunity by facilitating antigen presentation to T cells and subsequently activating them to target tumor cells. We compared the antigen presentation function of memory B cells in non-obese tumor samples and obese tumor samples. Surprisingly, our findings indicated that the antigen presentation capacity of memory B cells was lower in obese CRC compared to non-obese CRC (Fig. 5H). In summary, our findings highlight the significant involvement of endothelial cells in the recruitment of IgG^+^plasma cells in obese CRC. Concurrently, we observed a diminished antigen presentation capacity of memory B cells in obese CRC, thereby exacerbating the development of an immunosuppressive microenvironment.

### Characteristics of cancer cells in obese CRC

**Fig. 6:**
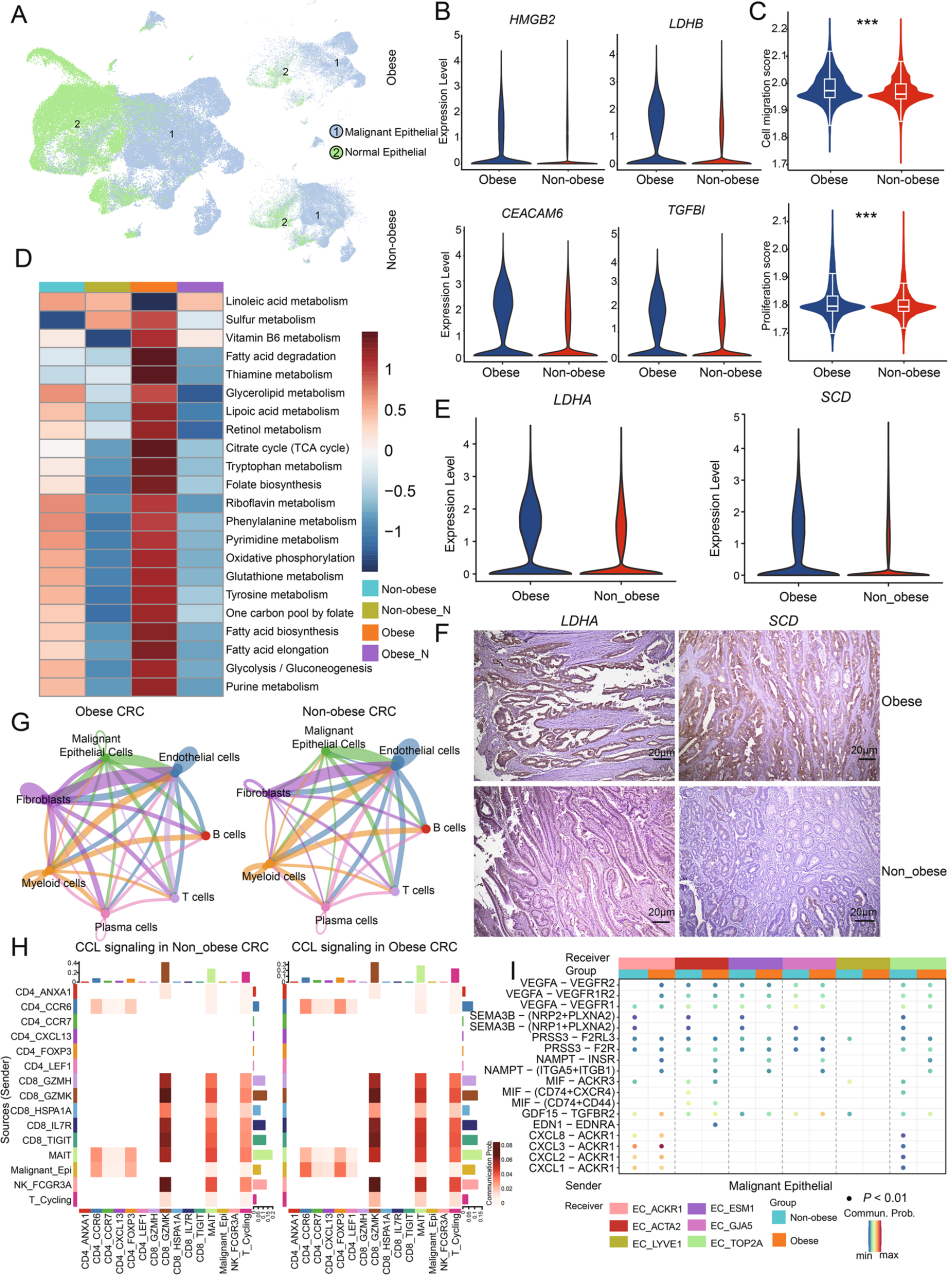
Characteristics of cancer cells in obese CRC. **A** UMAP plots of the epithelial cell, colored according to inferCNV inferred results. **B** Violin plots showing the expression of DEGs in malignant epithelial cells, colored by groups. **C** Violin plots showing the invasive characteristics score in malignant epithelial cells. Comparisons were performed by unpaired two-tailed Student’s t-test. Significance levels are expressed as *P < 0.05, **P < 0.01 and ***P < 0.001. **D** Heatmap showing the metabolic activity score of malignant epithelial cells by GSVA. The color darkness represents the scaled metabolic score. **E** Violin plots showing the expression of metabolism related DEGs in malignant epithelial cells, colored by groups. **F** Representative images of IHC staining detecting *LDHA* and *SCD* expression in obese CRC and non-obese samples. Scale bar, 20 μm. **G** The number of intercellular communications among different cell types in obese CRC and non-obese CRC samples. The line color represents cell types, and the line thickness represents interaction numbers. **H** Heatmap showing the overall CCL signaling by aggregating outgoing and incoming signaling together in the obese CRC and Non-obese CRC groups. **I** Dot plots showing the comparison of communication probabilities from malignant epithelial cells to six endothelial cell subsets among different groups

We distinguished malignant from nonmalignant epithelial cells based on the chromosomal profiles inferred from the scRNA-seq data (Fig. 6A, Additional file 8: Fig. S6A). Differentially expressed genes between obese CRC and non-obese CRC malignant epithelial cells have been identified (Fig. 6B, Additional file 8: Fig. S6B). The expression of genes involved in glycolysis metabolism, such as *HMGB2* and *LDHB*, was higher in obese CRC than in non-obese CRC samples (Fig. 6B). In addition, some genes associated with poor prognosis of CRC, such as *CEACAM6* and *TGFBI*, were also highly expressed in obese CRC samples (Fig. 6B). Previous studies have reported that overexpression of *CEACAM6* in a variety of malignant tumors can promote cell invasion and metastasis [51], and *TGFBI* promotes tumorigenesis and development by increasing cancer cell chemotaxis and cell migration potential of a variety of cancer cell types in vitro and in vivo [52]. Therefore, we further explored the feature scores associated with metastasis in obese CRC and non-obese CRC malignant epithelial cells. The results showed that malignant epithelial cells in obese CRC had higher scores of cell migration and proliferation characteristics than those in non-obese CRC (Fig. 6C). These results indicate that obese CRC has a higher invasive potential, which is consistent with clinical observations.

In order to investigate potential differences in malignant epithelial cells metabolism between obese CRC and non-obese CRC samples, we conducted an analysis of metabolic pathway activity in each malignant epithelial cell and identified pathways that were specifically activated in obese CRC (Fig. 6D). The results revealed that pathways related to glycolysis and fatty acid metabolism, including fatty acid biosynthesis and fatty acid elongation, exhibited significantly higher activity in obese CRC malignant epithelial cells (Fig. 6D). Notably, *LDHA*, a key gene involved in glycolysis, was found to be significantly up-regulated in obese CRC. Furthermore, *SCD*, an enzyme that controls synthesis of unsaturated fatty acids, as essential in breast and prostate cancer cells [53], was also observed to be elevated in obese CRC (Fig. 6E). More importantly, IHC staining further verified at the clinical CRC sample scale that the density of *LDHA* and *SCD* was increased in tumor regions of obese CRC samples compared to the non-obese CRC samples (Fig. 6F, Additional file 8: Fig. S6C). Recent research has reported increased uptake of fatty acids by tumor cells, while tumor-infiltrating CD8^+^T cells remain unaffected in high-fat diet (HFD)-induced obese mice. These different adaptations result in altered fatty acid distribution within HFD tumors, leading to impaired infiltration and function of CD8^+^T cells [22]. This suggests that tumor cells in obese CRC may competitively consume metabolic substrates that are needed by T cells, consequently impairing T cell function.

We analyzed intercellular communication between different cell types in both obese and non-obese CRC samples. The results indicated that the interactions between malignant epithelial cells and ECs were the most abundant in both obese and non-obese CRC samples (Fig. 6G). The number and strength of cell-to-cell interactions were higher in obese CRC samples compared to non-obese CRC samples (Additional file 8: Fig. S6D). Additionally, the obese CRC samples exhibited a higher frequency of interactions between malignant epithelial cells and T cells as well as ECs in comparison with that in the non-obese CRC samples (Additional file 8: Fig. S6E). We then examined the interactions between malignant epithelial cells and T cells in both non-obese and obese CRC samples, the results revealed that the CCL pathway displays extensive interactions (Fig. 6H). Specifically, in obese CRC samples, the CCL pathway displayed enhanced interactions compared to non-obese CRC samples. These interactions were notably observed between malignant epithelial cells and Treg cells, as well as between malignant epithelial cells and CD4^+^CCR6^+^T cells (Fig. 6H). We further identified specific ligand receptors for the CCL pathway between malignant epithelial cells and T cell subpopulations. Notably, the interaction between the chemokine *CCL20* and its receptor *CCR6* was significantly activated from malignant epithelial cells to Treg cells and CD4^+^CCR6^+^T cells of obese CRC samples (Additional file 8: Fig. S6F). Previous studies have reported that the chemokine *CCL20* facilitates the recruitment and retention of CCR6^+^Treg cells, thereby promoting tumor invasion and progression [54]. In the analysis of interactions between malignant epithelial cells and different subsets of ECs in both non-obese CRC samples and obese CRC samples, it was found that the chemokine *CXCL3* and its receptor *ACKR1* exhibited more pronounced activation from malignant epithelial cells towards ACKR1^+^EC in obese CRC samples compared to non-obese CRC samples (Fig. 6I). The interaction of malignant epithelial cells with T cells and ECs suggests that chemokines play an important role in cell interactions in obese CRC. Considering the potential of obesity to induce chronic inflammation, we next evaluated the expression of malignant epithelial cells chemokines in both obese and non-obese CRC samples. Remarkably, *CXCL3*, the ligand for *ACKR1*, exhibited the highest expression levels within malignant epithelial cells of obese CRC samples (Additional file 8: Fig. S6G). The results showed significantly higher expression of chemokines in obese CRC compared to non-obese CRC, suggesting that malignant epithelial cells in obese CRC might play a critical role in promoting the formation of an inflammatory microenvironment.

In conclusion, our results showed that glycolysis metabolism and fatty acid metabolism were significantly enhanced in malignant epithelial cells of obese CRC, while communication between malignant epithelial cells and T cells may be involved in the immunosuppression in obese CRC.

## Discussion

Compared with non-obese CRC, overweight and obese CRC is more aggressive and has a worse prognosis [8–10]. At the same time, the incidence of obesity-related CRC is increasing year by year. Therefore, there is an urgent need to characterize the cellular and molecular mechanisms of overweight and obese CRC to clarify the mechanism by which obesity increases tumor invasiveness and to identify new therapeutic targets to improve the prognosis of obese CRC patients. Previous studies have found that obesity drives epigenomic changes in the colon epithelium and reprograms cell metabolism in the colon epithelium to promote CRC tumor initiation and progression. However, the most of previous studies on obesity and CRC only focused on tumor-intrinsic effects or on the endocrine-tumor cell regulatory axis. It has not yet been reported how changes in the tumor microenvironment in overweight/obese CRC.

To the best of our knowledge, this is the first study to investigate the characteristics of the tumor microenvironment in overweight/obese CRC at the single-cell resolution level. Our study investigated the characteristics of tumor cells and the immune microenvironment in obese CRC. Based on single-cell level analysis, we found that effector T cells from obese CRC samples were less cytotoxic than those from non-obese CRC samples while having lower glycolysis metabolic activity. Interestingly, we observed that the glycolysis metabolic activity of the tumor cells was higher in obese CRC compared to non-obese CRC samples. This led us to speculate that tumor cells may compete for the metabolites essential for T cell function, thus contributing to the dysfunction of effector T cells in obese CRC samples. Furthermore, we found that the antigen-presenting capacity of B cells and DCs, which play a pivotal role in T cell activation, was diminished in obese CRC samples. This further exacerbates the dysfunction of effector T cells in obese CRC. Additionally, our cell interaction analysis revealed that tumor cells in obese CRC samples may recruit Treg cells through the *CCL20-CCR6* axis, thereby enhancing the immunosuppressive function of Treg cells. Our study also demonstrated a more pronounced depleted phenotype of CD4^+^T cells in obese CRC samples, characterized by high expression of immune checkpoint molecules such as *CTLA4* and *TIGIT*. Interestingly, we observed higher expression of *PDCD1* in CXCL13^+^CD4^+^T cells in obese CRC samples. Some previous studies have reported that overweight or obese patients with various types of cancers, including melanoma, non-small cell lung cancer, and renal cell cancer, showed better responses to anti-PD-1/PD-L1 treatment [55]. There have also been reports on the impact of obesity on CTLA-4 treatment, where patients with BMI ≥ 25 (overweight or obese) had significantly improved response rates compared to patients with BMI < 25 (normal or underweight) who received ipilimumab as monotherapy for metastatic melanoma [56]. Therefore, based on our data, we speculate that overweight/obese CRC may also have better immune checkpoint response, and the underlying mechanism may be related to the higher expression of immune checkpoint molecules in obese CRC. In addition, our findings indicate that metabolic dysfunction in NK cells contributes to the impairment of their cytotoxic process in obese CRC samples, suggesting that obesity hampers the anti-tumor response of NK cells through metabolic reprogramming. Interestingly, our data also revealed significantly higher expression of the inhibitory receptor *SIGLEC7* in NK cells from obese CRC samples. Recent reports have emphasized the immunomodulatory role of Siglecs in the anti-tumor response of T cells and NK cells [57]. The elevated expression of *SIGLEC7* has been shown to diminish the cytotoxic activity of NK cells against bladder cancer cells [42]. According to our study, *SIGLEC7* may serve as a potential immune checkpoint in overweight/obese CRC. Moreover, recent experiments conducted in a humanized immunocompetent mouse model demonstrated that blocking *SIGLEC7* or *SIGLEC9* reduces tumor burden in vivo, providing support for the use of anti-SIGLEC antibodies as a means to enhance anti-tumor immunity [58]. Therefore, targeting *SIGLEC7* in NK cells holds promise as a potential treatment strategy for overweight/obese CRC.

Differences between obese CRC and non-obese CRC can lead to differences in tumor characteristics and progression, especially in immune response and metabolic pathways. TAMs are important tumor-promoting cells. Numerous previous studies have shown that TAMs have been shown to regulate many key processes related to malignant tumors, including promoting tumor cell growth, drug resistance, metastasis, inducing angiogenesis and immunosuppression [59]. Using the scMetabolism package, we found that the 3 LAMs cell subset in obese CRC samples exhibited the highest metabolic activity, especially in lipid metabolism. Previous studies have also highlighted the importance of lipid metabolism in the differentiation and function of tumoral TAM in TME [59]. Analysis of DCs revealed that antigen presentation function was generally reduced in obese CRC samples, which further impaired T cell activation. Surprisingly, our exploration of B cells also revealed a down-regulation of antigen presentation by memory B cells in obese CRC samples. Similar studies have previously shown disrupted priming and antigen presentation by dendritic cells to T cells has also been reported in obesity [60]. In addition, it has been found that in the context of obesity, DC-dependent immunotherapy is ineffective against renal cell carcinoma in obese mice but induces a response in normal-weight mice [61]. Taken together, these results emphasize that obesity leads to immune dysfunction.

Our findings also highlight the potential tumor promoting role of stromal cells in obese CRC. Obesity is characterized by chronic inflammation due to immune cell infiltration and fibrosis due to ECM dysregulation [62]. Myofibroblasts in obese CRC samples show a greater capacity for ECM remodeling, whereas fibrotic ECM stimulates mechanical signaling in surrounding cancer cells, thereby promoting cancer growth [63]. The pseudotime analysis of fibroblasts highlighted the role of hypoxic pathways during myofibroblast activation, so we hypothesized that the greater capacity of myofibroblasts for ECM remodeling in obese CRC samples might be related to TME hypoxia in obese CRC. Previous reports have highlighted the critical role of hypoxia-inducible factor (HIF)-1α in the pathogenesis of fibrosis through ECM remodeling [64]. Within the EC subgroup, we found more ESM1^+^EC enrichment in obese CRC samples, and its abundance was negatively correlated with the survival of CRC patients. Functional enrichment analysis of EC subclusters demonstrated that their potential tumor-promoting role in TME may be related to the migration and adhesion of epithelial cells.

Furthermore, our study also revealed that tumor cells in obese CRC samples exhibit more aggressive behavior, including heightened cell migration and proliferation abilities compared to non-obese CRC. Analysis of metabolic pathways showed that glycolysis and fatty acid metabolic pathways were significantly more active in obese CRC than in non-obese CRC samples. Metabolic reprogramming, a prominent hallmark of cancer, plays a crucial role in facilitating the initiation and progression of malignant tumors, with glycolysis being a key feature of this reprogramming [65]. In the context of limited blood supply in solid tumors, the high nutrient consumption by tumor cells may impede the metabolic demands of intratumoral T cells. Recent research has highlighted that the presence of obesity in tumor cells can lead to T-cell dysfunction due to altered fatty acid allocation and localized depletion of essential metabolites [22]. Based on this, we proposed that the heightened glycolysis of tumor cells in obese CRC may competitively deplete metabolites necessary for effector T cell function, potentially resulting in the down-regulation of their functionality. Examination of various cell types within the TME indicated that the TME of obese CRC was in an inflammatory state, with tumor cells engaging in extensive intercellular interactions with other cells via chemokines.

## Conclusions

Our study found that overweight/obese CRC has a more immunosuppressive microenvironment and distinct metabolic reprogramming characterized by increased of glycolysis and lipid metabolism at the single-cell level. These changes may affect modified nutrient availability and immune dysfunction within the tumor microenvironment. This provides valuable insights for the potential treatment strategies for overweight/obese CRC patients.

### Supplementary Information

**Additional file 1 Table S1.** Clinical characteristics of the patients.

**Additional file 2: Table S2.** Gene list of signatures for GSVA.

**Additional file 3: Figure S1.** Clustering and tissue distribution of major cell types. A UMAP plot of the cells types, colored by samples. B Tissue prevalence of each cell types estimated by Ro/e score, in which Ro/e denotes the ratio of observed to expected cell number.

**Additional file 4: Figure S2.** Characterization of T cell subsets. A Dot plots showing the expression of the marker genes in T cells. The pct.exp reflects the percentage of cells expressing the gene at non-zero levels. The Average expression reflects the averaged log-normalized expression. B Violin plots showing the cytotoxic score in five CD8+T cell subsets. C Violin plots showing PDCD1 expression of CD4+CXCL13+T cells from obese CRC and non-obese CRC samples.

**Additional file 5: Figure S3.** Myeloid cells analysis. A Heatmap showing the macrophage characteristic score by GSVA. The color darkness represents the scaled metabolic score. B The Spearman correlation analysis between the scores of cytotoxic score in GZMK+T cells and antigen presentation score in DCs in CRC samples.

**Additional file 6: Figure S4.** Characterization of stromal cells. A Tissue prevalence of each fibroblast subsets estimated by Ro/e score, in which Ro/e denotes the ratio of observed to expected cell number. B Heatmap showing the expression of canonical fibroblasts marker genes. C Two-dimensional plots showing the expression scores for genes related to hypoxia and TGFB signaling pathway, in fibroblasts, along with the pseudotime.

**Additional file 7: Figure S5**. Marker genes for B cells. A Dot plots showing the expression of the marker genes in B cells. The pct.exp reflects the percentage of cells expressing the gene at non-zero levels. The Average expression reflects the averaged log-normalized expression.

**Additional file 8: Figure S6.** Heterogeneity of cancer cells. A UMAP plots of the epithelial cell, colored by groups. B Volcano plot showing the DEGs of cancer cells in obese CRC and non-obese CRC. Red and blue represent DEGs that are up- and down-regulated in obese CRC, respectively. C Boxplot indicating the IHC average optical density of LDHA and SCD in obese CRC (n = 20) and non-obese (n = 20) samples. Comparisons were performed by unpaired two-tailed Student’s t-test. Significance levels are expressed as *P <0.05, **P <0.01 and ***P <0.001. D Bar plots showing the number and strength of cell interactions in all cell types. E The number of intercellular communications among different cell types in obese CRC and non-obese CRC samples. The line color represents cell types, and the line thickness represents interaction numbers. F Dot plots showing the comparison of communication probabilities from malignant epithelial cells to T cell subsets among different groups. G Dot plots showing the expression of the chemokine in malignant epithelial cells. The pct.exp reflects the percentage of cells expressing the gene at non-zero levels. The Average expression reflects the averaged log-normalized expression.

## Data Availability

The datasets utilized and/or analyzed during the current study can be obtained from the corresponding author upon reasonable request.

## Abbreviations

CRC: Colorectal cancer
TME: Tumor microenvironment
scRNA-seq: Single-cell RNA sequencing
BMI: Body mass index
DPBS: Dulbecco’s phosphate-buffered saline
DEGs: Differentially expressed genes
GO: Gene ontology
GSVA: Gene set variation analysis
GSEA: Gene Set Enrichment Analysis
TCGA: The cancer genome atlas
SIGLECS: Sialic acid-binding immunoglobulin-like lectins
DCs: Dendritic cells
TAMs: Tumor-associated macrophages
LAMs: Lipid-associated macrophages
pDCs: Plasmacytoid dendritic cells
CAFs: Cancer-associated fibroblasts
NFs: Normal fibroblasts
ECM: Extracellular matrix
ECs: Endothelial cells
HFD: High-fat diet
IHC: Immunohistochemistry
