# Supplementary material for: Single-cell transcriptome analysis reveals immunosuppressive landscape in overweight and obese colorectal cancer
Source: J Transl Med. 2024 Feb 4;22:134. doi: 10.1186/s12967-024-04921-5 (PMC10838453; doi:10.1186/s12967-024-04921-5)
Supplement: Supplementary file 3 — Additional file 3: Figure S1. Clustering and tissue distribution of major cell types. A UMAP plot of the cells types, colored by samples. B Tissue prevalence of each cell types estimated by Ro/e score, in which Ro/e denotes the ratio of observed to expected cell number. [file 12967_2024_4921_MOESM3_ESM.pdf]

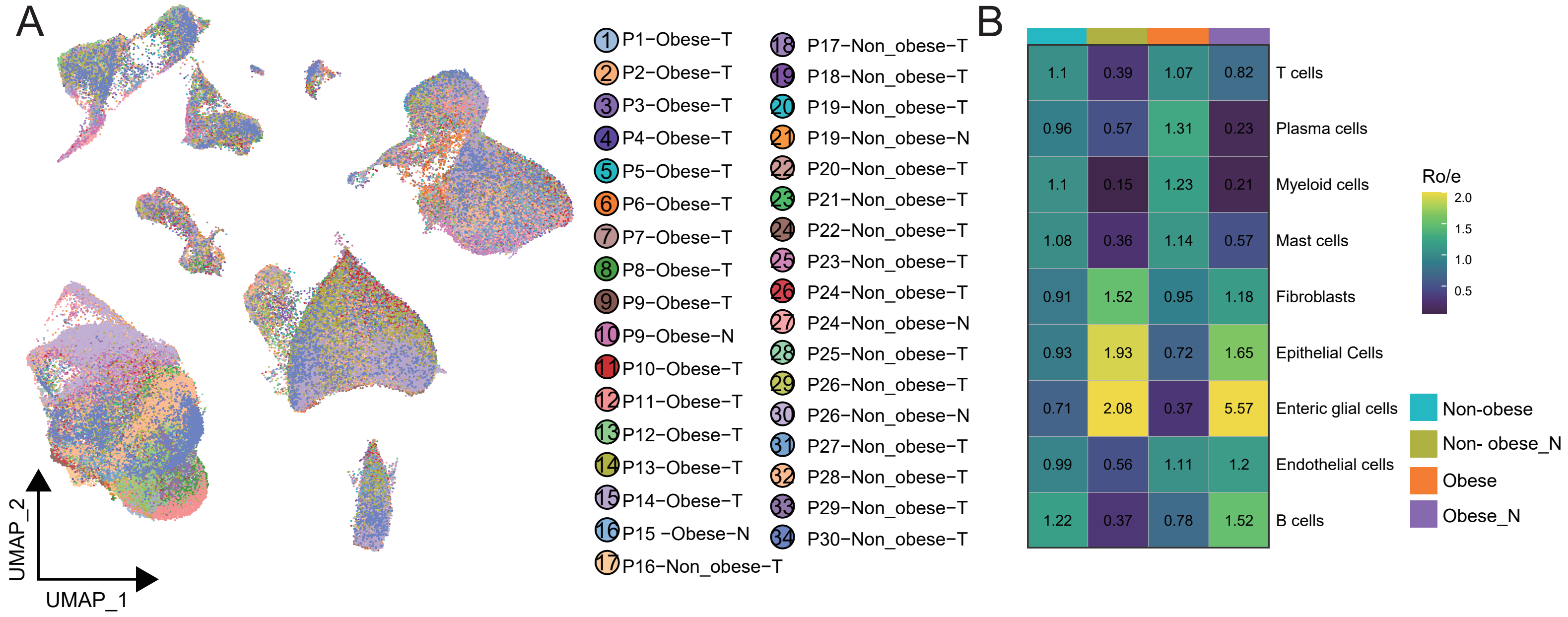

A
UMAP_2
UMAP_1
1 P1−Obese−T
2 P2−Obese−T
3 P3−Obese−T
4 P4−Obese−T
5 P5−Obese−T
6 P6−Obese−T
7 P7−Obese−T
8 P8−Obese−T
9 P9−Obese−T
10 P9−Obese−N
11 P10−Obese−T
12 P11−Obese−T
13 P12−Obese−T
14 P13−Obese−T
15 P14−Obese−T
16 P15 −Obese−N
17 P16−Non_obese−T
18 P17−Non_obese−T
19 P18−Non_obese−T
20 P19−Non_obese−T
21 P19−Non_obese−N
22 P20−Non_obese−T
23 P21−Non_obese−T
24 P22−Non_obese−T
25 P23−Non_obese−T
26 P24−Non_obese−T
27 P24−Non_obese−N
28 P25−Non_obese−T
29 P26−Non_obese−T
30 P26−Non_obese−N
31 P27−Non_obese−T
32 P28−Non_obese−T
33 P29−Non_obese−T
34 P30−Non_obese−T
B
T cells 1.1 0.39 1.07 0.82
Plasma cells 0.96 0.57 1.31 0.23
Myeloid cells 1.1 0.15 1.23 0.21
Mast cells 1.08 0.36 1.14 0.57
Fibroblasts 0.91 1.52 0.95 1.18
Epithelial Cells 0.93 1.93 0.72 1.65
Enteric glial cells 0.71 2.08 0.37 5.57
Endothelial cells 0.99 0.56 1.11 1.2
B cells 1.22 0.37 0.78 1.52
Ro/e
2.0
1.5
1.0
0.5
Non-obese
Non- obese_N
Obese
Obese_N
