# Supplementary figures and images for: Single-cell transcriptome analysis reveals immunosuppressive landscape in overweight and obese colorectal cancer
Source: J Transl Med. 2024 Feb 4;22:134. doi: 10.1186/s12967-024-04921-5 (PMC10838453; doi:10.1186/s12967-024-04921-5)

A

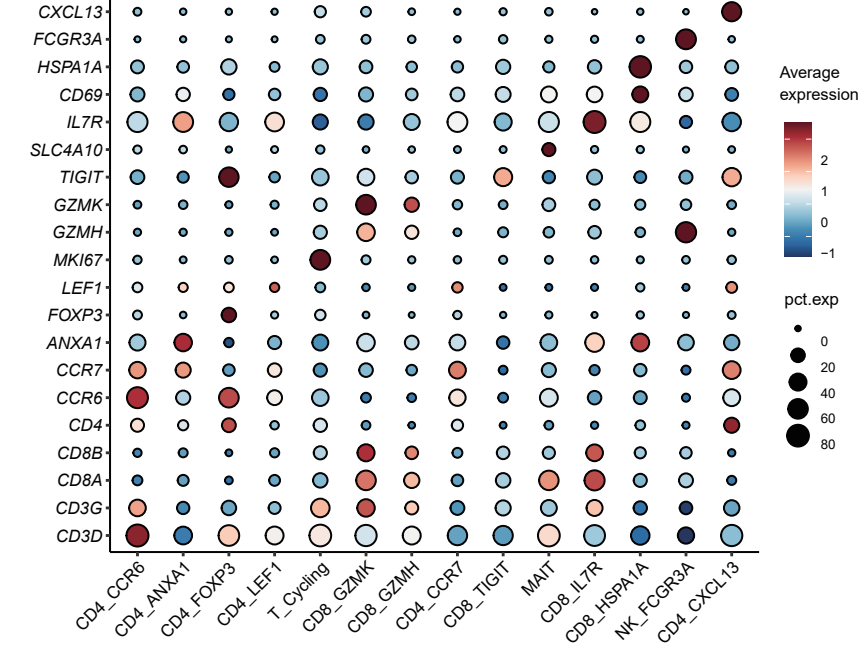

B

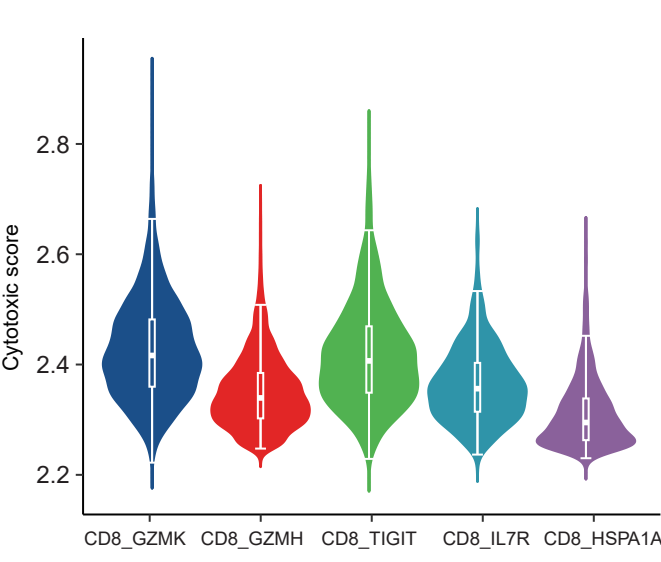

C

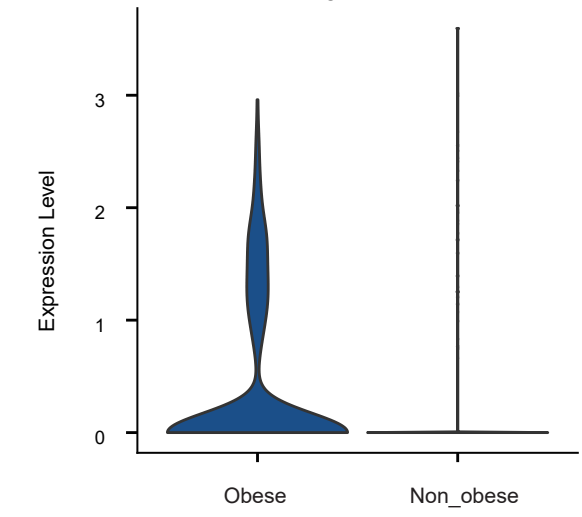

Supplement: Supplementary file 4 — Additional file 4: Figure S2. Characterization of T cell subsets. A Dot plots showing the expression of the marker genes in T cells. The pct.exp reflects the percentage of cells expressing the gene at non-zero levels. The Average expression reflects the averaged log-normalized expression. B Violin plots showing the cytotoxic score in five CD8+T cell subsets. C Violin plots showing PDCD1 expression of CD4+CXCL13+T cells from obese CRC and non-obese CRC samples. [file 12967_2024_4921_MOESM4_ESM.pdf]

A

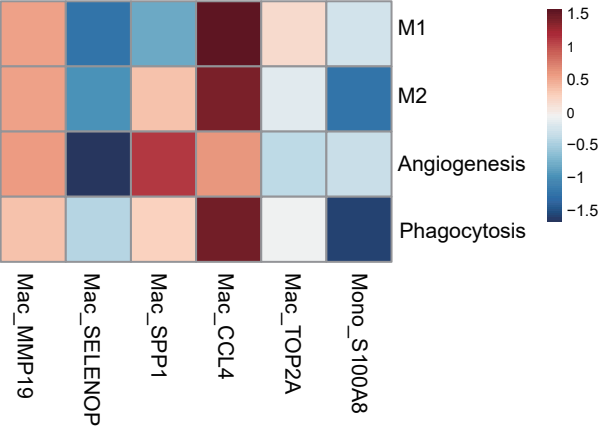

B

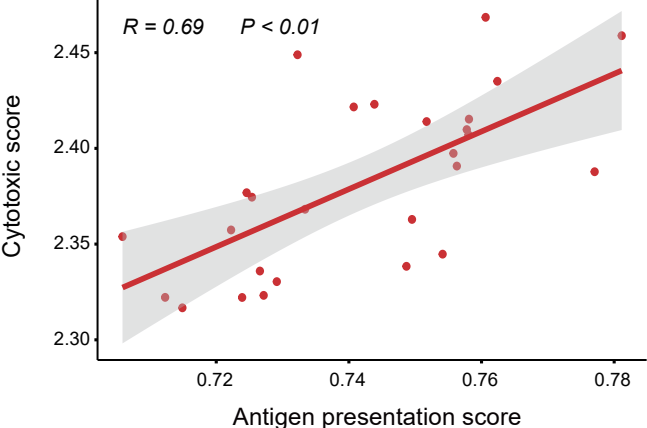

Supplement: Supplementary file 5 — Additional file 5: Figure S3. Myeloid cells analysis. A Heatmap showing the macrophage characteristic score by GSVA. The color darkness represents the scaled metabolic score. B The Spearman correlation analysis between the scores of cytotoxic score in GZMK+T cells and antigen presentation score in DCs in CRC samples. [file 12967_2024_4921_MOESM5_ESM.pdf]

A

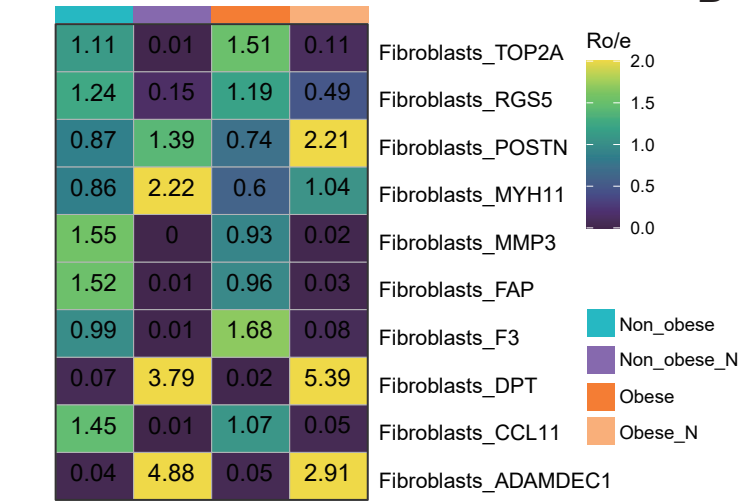

C

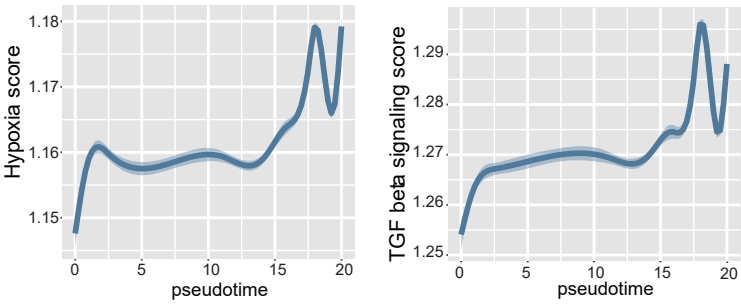

B

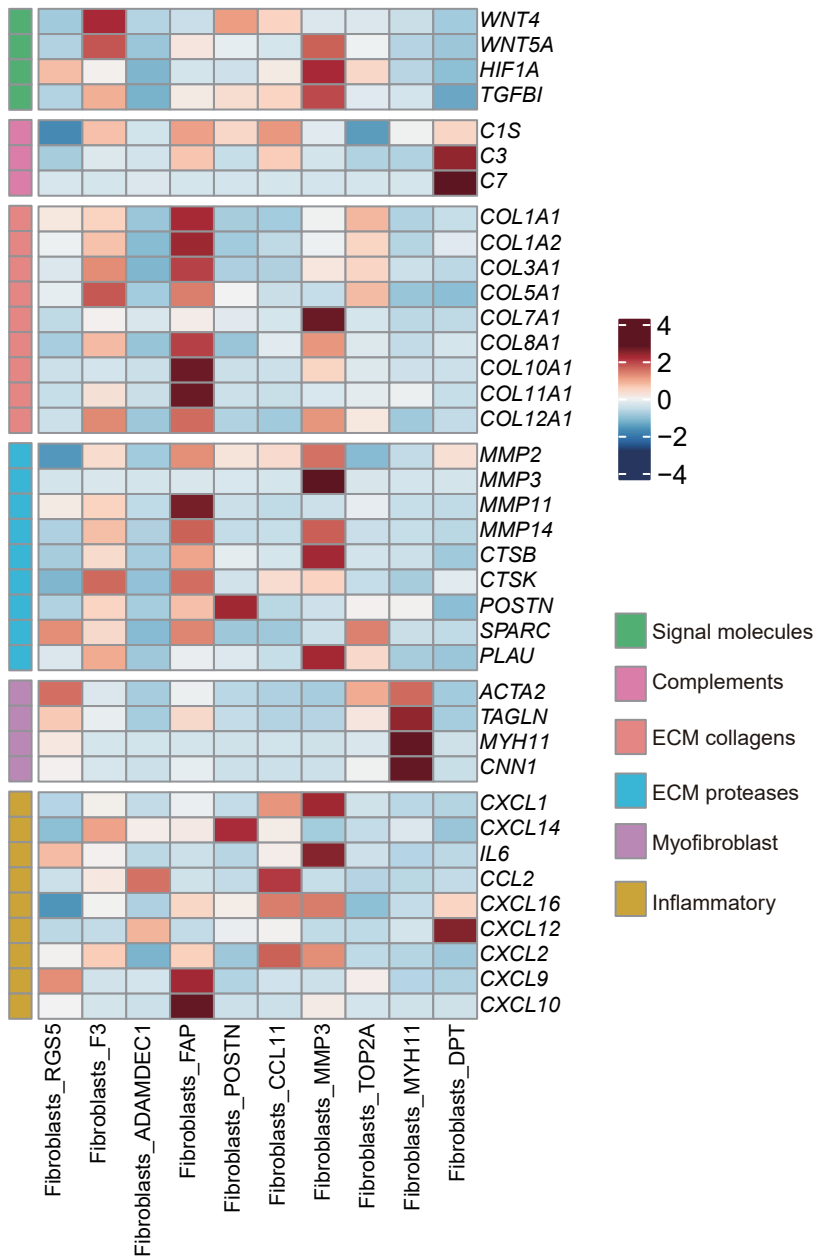

Supplement: Supplementary file 6 — Additional file 6: Figure S4. Characterization of stromal cells. A Tissue prevalence of each fibroblast subsets estimated by Ro/e score, in which Ro/e denotes the ratio of observed to expected cell number. B Heatmap showing the expression of canonical fibroblasts marker genes. C Two-dimensional plots showing the expression scores for genes related to hypoxia and TGFB signaling pathway, in fibroblasts, along with the pseudotime. [file 12967_2024_4921_MOESM6_ESM.pdf]

A

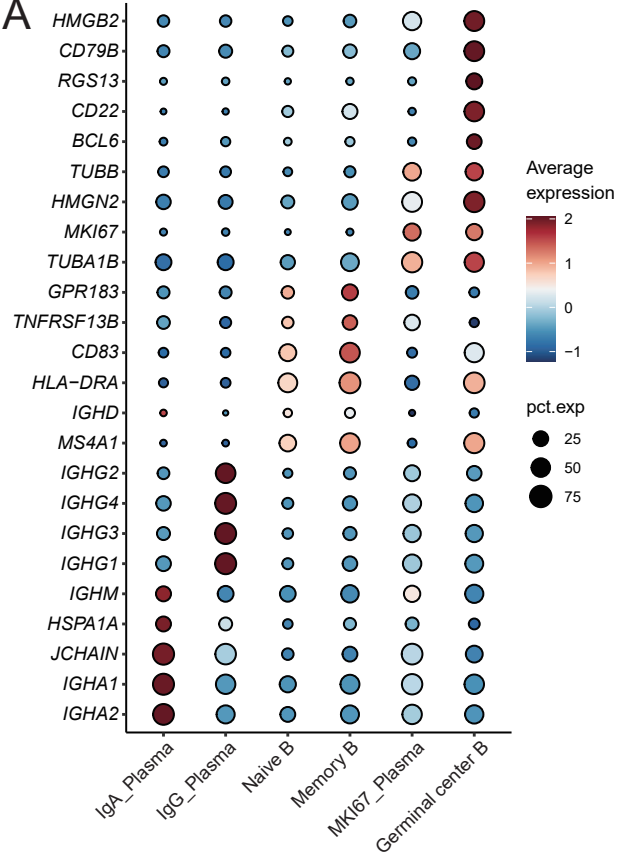

Supplement: Supplementary file 7 — Additional file 7: Figure S5. Marker genes for B cells. A Dot plots showing the expression of the marker genes in B cells. The pct.exp reflects the percentage of cells expressing the gene at non-zero levels. The Average expression reflects the averaged log-normalized expression. [file 12967_2024_4921_MOESM7_ESM.pdf]

A

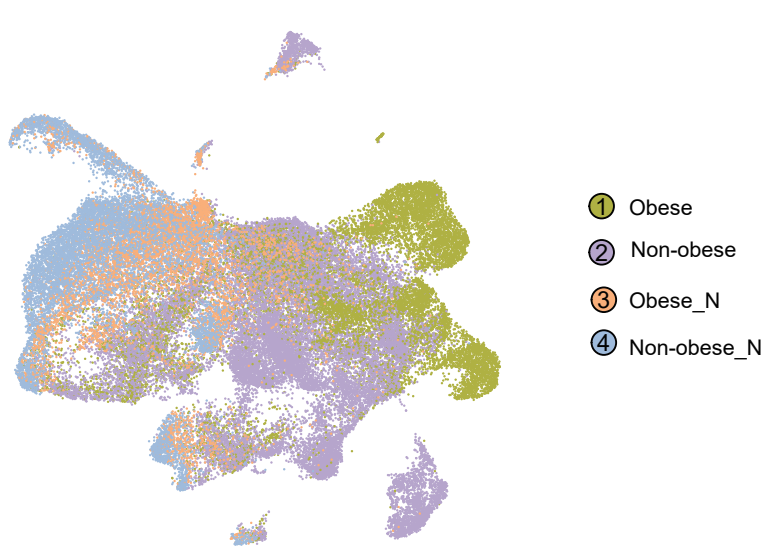

B

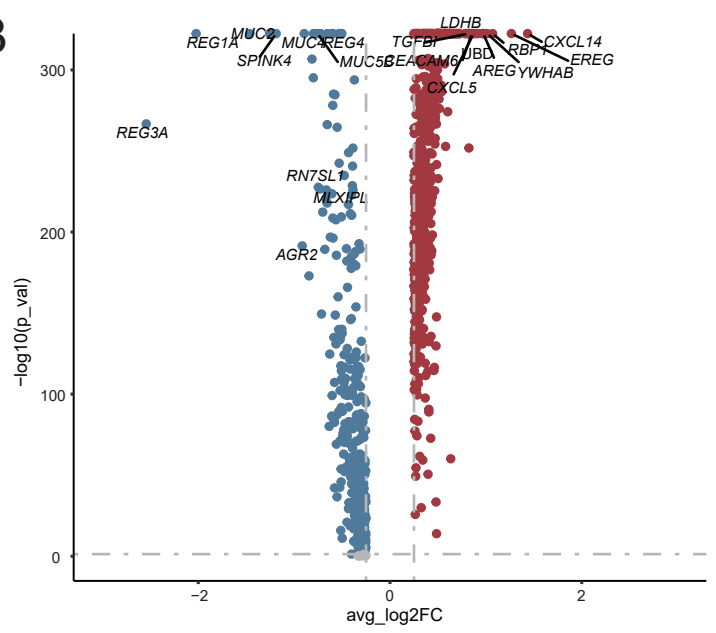

C

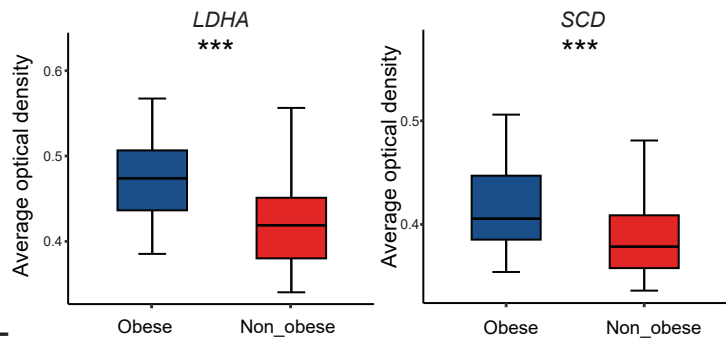

D

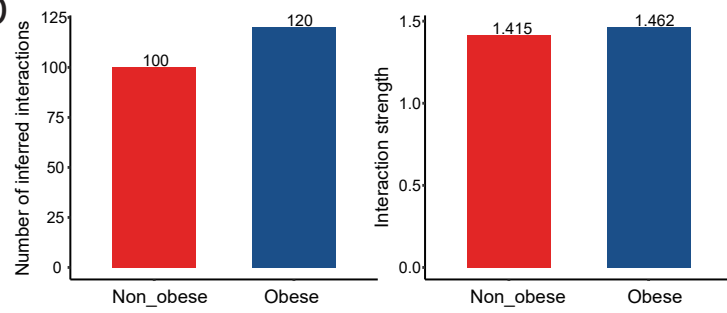

E

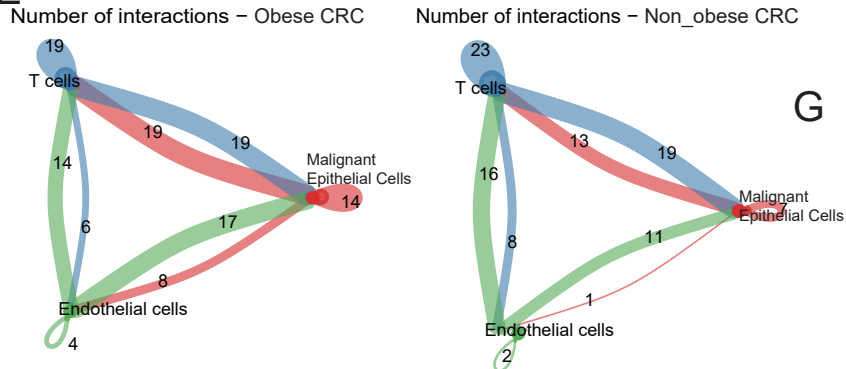

F

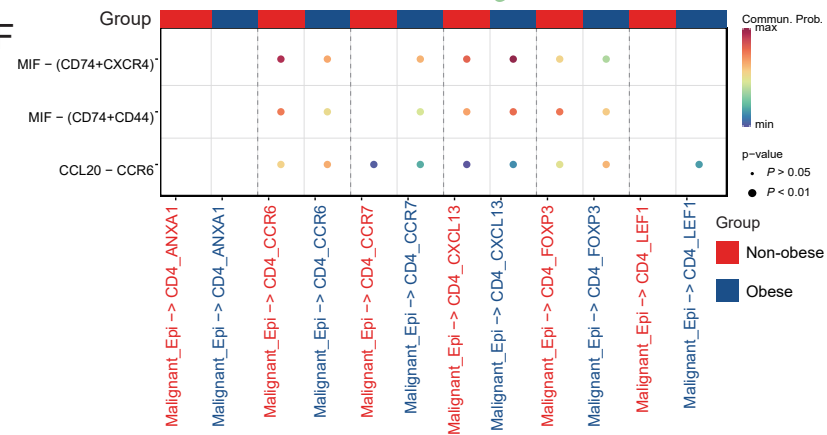

G

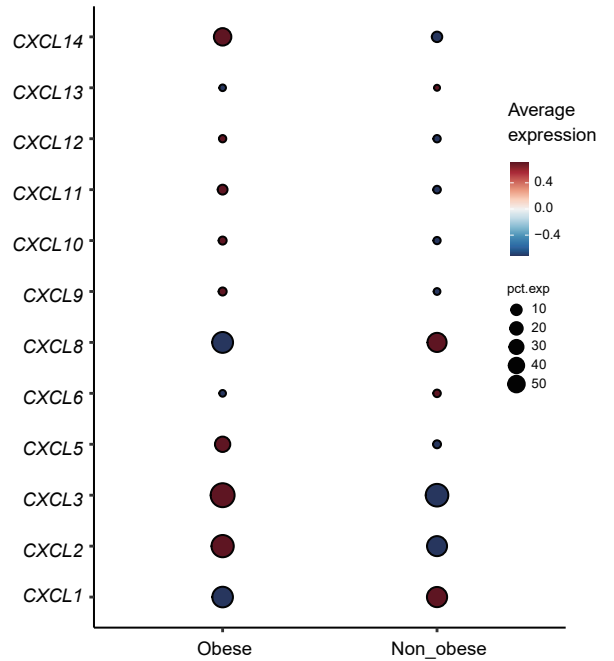

Supplement: Supplementary file 8 — Additional file 8: Figure S6. Heterogeneity of cancer cells. A UMAP plots of the epithelial cell, colored by groups. B Volcano plot showing the DEGs of cancer cells in obese CRC and non-obese CRC. Red and blue represent DEGs that are up- and down-regulated in obese CRC, respectively. C Boxplot indicating the IHC average optical density of LDHA and SCD in obese CRC (n = 20) and non-obese (n = 20) samples. Comparisons were performed by unpaired two-tailed Student’s t-test. Significance levels are expressed as *P <0.05, **P <0.01 and ***P <0.001. D Bar plots showing the number and strength of cell interactions in all cell types. E The number of intercellular communications among different cell types in obese CRC and non-obese CRC samples. The line color represents cell types, and the line thickness represents interaction numbers. F Dot plots showing the comparison of communication probabilities from malignant epithelial cells to T cell subsets among different groups. G Dot plots showing the expression of the chemokine in malignant epithelial cells. The pct.exp reflects the percentage of cells expressing the gene at non-zero levels. The Average expression reflects the averaged log-normalized expression. [file 12967_2024_4921_MOESM8_ESM.pdf]
